# Revealing Edible Bird Nest as Novel Functional Foods in Combating Metabolic Syndrome: Comprehensive In Silico, In Vitro, and In Vivo Studies

**DOI:** 10.3390/nu15183886

**Published:** 2023-09-06

**Authors:** Happy Kurnia Permatasari, Queen Intan Permatasari, Nurpudji Astuti Taslim, Dionysius Subali, Rudy Kurniawan, Reggie Surya, Faqrizal Ria Qhabibi, Melvin Junior Tanner, Siti Chairiyah Batubara, Nelly Mayulu, William Ben Gunawan, Andi Yasmin Syauki, Netty Salindeho, Moon Nyeo Park, Juan Alessandro Jeremis Maruli Nura Lele, Raymond R. Tjandrawinata, Bonglee Kim, Fahrul Nurkolis

**Affiliations:** 1 Department of Biochemistry and Biomolecular, Faculty of Medicine, University of Brawijaya, Malang 65145, Indonesia; 2 Department of Pharmacy, Faculty of Medicine, University of Brawijaya, Malang 65145, Indonesia; 3 Division of Clinical Nutrition, Department of Nutrition, Faculty of Medicine, Hasanuddin University, Makassar 90245, Indonesia; 4 Department of Biotechnology, Faculty of Biotechnology, Atma Jaya Catholic University of Indonesia, Jakarta 12930, Indonesia; 5 Diabetes Connection Care, Eka Hospital Bumi Serpong Damai, Tangerang 15321, Indonesia; 6 Department of Food Technology, Faculty of Engineering, Bina Nusantara University, Jakarta 11480, Indonesia; 7 Medical School Department, Faculty of Medicine, Brawijaya University, Malang 65145, Indonesia; 8 Nutrition Coaching Development, PT. Prima Sehat Makmur Utama, Jakarta 12430, Indonesia; 9 Food Technology Department, Sahid University of Jakarta, South Jakarta 12870, Indonesia; 10 Department of Nutrition, Faculty of Health Science, Muhammadiyah Manado University, Manado 95249, Indonesia; 11 Department of Nutrition Science, Faculty of Medicine, Diponegoro University, Semarang 50275, Indonesia; 12 Fishery Products Technology Study Program, Faculty of Fisheries and Marine Sciences, Sam Ratulangi University, Manado 95115, Indonesia; 13 Department of Pathology, College of Korean Medicine, Kyung Hee University, Seoul 02447, Republic of Koreabongleekim@khu.ac.kr (B.K.); 14 Korean Medicine-Based Drug Repositioning Cancer Research Center, College of Korean Medicine, Kyung Hee University, Seoul 02447, Republic of Korea; 15 Faculty of Medicine, Universitas Kristen Indonesia, Jakarta 13630, Indonesia; 16 Dexa Laboratories of Biomolecular Science, Dexa Medica Group, Cikarang 17530, Indonesia; 17 Department of Biological Sciences, Faculty of Sciences and Technology, State Islamic University of Sunan Kalijaga (UIN Sunan Kalijaga), Yogyakarta 55281, Indonesia; fahrul.nurkolis.mail@gmail.com

**Keywords:** edible bird nest, swallow bird nest, metabolic syndrome, functional food, terpenoids, metabolites, antioxidants, antiobesity, antidiabetic

## Abstract

Metabolic dysfunction, which includes intra-abdominal adiposity, glucose intolerance, insulin resistance, dyslipidemia, and hypertension, manifests into metabolic syndrome and related diseases. Therefore, the discovery of new therapies in the fight against metabolic syndrome is very challenging. This study aims to reveal the existence of an edible bird nest (EBN) as a functional food candidate that may be a new alternative in fighting metabolic syndrome. The study included three approaches: in silico molecular docking simulation, in vitro, and in vivo in rats fed on cholesterol- and fat-enriched diets. Four terpenoids of Bakuchiol, Curculigosaponin A, Dehydrolindestrenolide, and 1-methyl-3-(1-methyl-ethyl)-benzene in EBN have been identified through LCMS/MS-QTOF. In molecular docking simulations, Bakuchiol and Dehydrolindestrenolide are considered very potent because they have higher inhibitory power on the four receptors (iNOS, ROS1 kinase, FTO, and lipase) than standard drugs. In vitro tests also provide insight into the antioxidant, antidiabetic, and antiobesity activities of EBN, which is quite feasible due to the smaller EC_50_ value of EBN compared to standard drugs. Interestingly, in vivo studies also showed significant improvements (*p* < 0.05) in the lipid profile, blood glucose, enzymatic levels, and inflammatory biomarkers in rats given high-dose dietary supplementation of EBN. More interestingly, high-dose dietary supplementation of EBN upregulates PGC-1α and downregulates HMG-CoA reductase. Comprehensively, it has been revealed that EBN can be novel functional foods for combating metabolic syndrome.

## 1. Introduction

Metabolic syndrome is a cluster of interrelated risk factors that reflect overnutrition, sedentary lifestyles, and resultant excess adiposity [1]. It is characterized by a combination of risk factors encompassing obesity, high blood sugar levels, insulin resistance, elevated blood pressure, and non-HDL cholesterol [2,3]. It has been widely accepted that metabolic syndrome significantly increases the likelihood of developing cardiovascular diseases and diabetes mellitus [4]. About one-third of adults in the US have been reported to suffer from metabolic syndrome [2]. Concerning obesity as a risk factor for metabolic syndrome, the World Health Organization (WHO) stated that there were 650 million adults, 340 million adolescents, and 39 million children who were obese [5]. Today, obesity is not even considered a disease of affluence anymore, according to a global survey, since its prevalence has doubled in 73 countries and increased in other countries in the last quarter decade, most of which were countries with low socio-economic index [6]. Since environmental factors play an essential role in the development of metabolic syndrome, adopting a healthy lifestyle is paramount to preventing its prevalence [7]. Therefore, eating in a healthy manner could be adopted to support a healthy lifestyle that would prevent metabolic syndrome, such as by consuming functional food.

Previously, the use of functional foods has been suggested as a potential therapeutic option for treating metabolic syndrome, in particular with regard to its relation to obesity [8]. Edible bird nest (EBN), a renowned Asian delicacy derived from the saliva of swiftlets, has been consumed in many parts of the world for its nutritional and medical values [9], thus suggesting it to be a functional food. EBN is mainly produced in Southeast Asian countries (mainly Indonesia, Thailand, and Malaysia) and has been culturally regarded as a high-grade, expensive health food [10]. The nutritional content of EBN dry matter is mainly constituted of protein (>50%), followed by carbohydrates (40–45%) and ash (5%) with very little fat (<0.5%) [11]. Peptides are the most important protein components in EBN, with the total essential amino acids appreciably greater than in other foods known as sources of protein, such as eggs and milk [12]. Sialic acid is the major component of carbohydrates in EBN (10%) besides mannose, glucosamine, galactosamine, galactose, and fucose [13]. Studies focusing on metabolites such as terpenoids and their biological activity from EBN are limited to date and need further exploration.

Several scientific studies have been conducted to explore the potential of EBN for health promotion. Bioactive peptides and glycoproteins present in EBN are often considered to be the main compounds contributing to the health benefits of EBN [14]. Several recognized health properties of EBN include anti-influenza virus, immunomodulatory, antioxidant, anti-inflammatory, and anti-aging. In addition, EBN has been reported to improve neurodegenerative and cardiovascular diseases [9]. With regard to metabolic syndrome, EBN has been reported to prevent insulin resistance induced by a high-fat diet in rats [15] and ameliorate atherosclerosis in hypercholesterolemic mice [16]. Hydrolyzed bird nests had the potential to regulate pancreatic B-cell function and insulin signaling in diabetic mice [17]. 

These nutritional characteristics make EBN an intriguing subject for scientific exploration and potential therapeutic applications. Further research is warranted to elucidate the specific bioactive components and mechanisms underlying the potential health benefits associated with the consumption of EBN. The present study aimed to investigate the effects of EBN consumption on cholesterol- and fat-enriched diet animal models. The focus was emphasized on the lipid profile, blood glucose, enzymatic metabolic, and inflammatory biomarkers; more specifically, to complement the current evidence-based EBN, this study also looked at modulatory effects on peroxisome proliferator-activated receptor-gamma coactivator-1 alpha (PGC-1α) and HMG-CoA reductase. The PGC-1α pathway regulates cellular metabolism, energy homeostasis, and aging [18]. Dysregulation of this pathway is implicated in metabolic disorders and age-related diseases [18]. HMG-CoA reductase, as an enzyme regulating the rate of cholesterol biosynthesis, and HMG-CoA reductase inhibitors still need to be explored further to fight metabolic syndrome [18]. By knowing the modulation, there will be a new insight into the evidence base of EBN health benefits. 

In addition, the characterization of chemical constituents in EBN and their biological activities were also analyzed using computational molecular docking on the selected receptors. In vitro antioxidant, antidiabetic, and antiobesity activities were also reported in this study to complement the current EBN literature. Understanding the full potential of EBN as a functional food may provide valuable insights into its role in managing metabolic syndrome and improving overall health outcomes.

## 2. Materials and Methods

### 2.1. Edible Bird Nest

EBN was obtained by our research team from wallet bird farmers in Pasuruan Regency, East Java, Indonesia, as formally confirmed. The EBN obtained had gone through a cleaning process by certified farmers and was ready to eat. EBN did not go through any extraction process because it aims to see the benefits if consumed as usual (consumption of EBN by the community without any extraction treatment), as the researchers wanted to study the impact directly, which is similar to consumption habits by consumers. Because of their edible and resistant nature, EBN samples were stored in aluminum foil at room temperature before further laboratory tests.

### 2.2. Metabolites Screening by LCMS/MS-QTOF Analysis

EBN samples were sent to SIG Laboratory (ISO 17025:2017 Accredited LP-184-IDN, Bogor, West Java) for liquid chromatography analysis of mass spectrometry-quadrupole—time of flight (LCMS/MS-QTOF) with a GIS certificate number of LHP. VI.2023.051606162. LCMS/MS-QTOF analysis is performed according to Qiao et al. [19]. The sample preparation involves weighing 1 g of the sample into a 10 mL volumetric flask. Next, suitable methanol or solvent was added and sonicated for 30 min. The mixture was concentrated with suitable methanol or solvent and homogenized. The mixture was then filtered through a GHP/PTFE 0.22-μm membrane filter and injected into the UPLC system. The instrumental measurement conditions were set as follows: LC Column = C18, Column Temperature = 40 °C, Autosampler Temperature = 15 °C, Injection Volume = 10 μL, Mobile Phase = A = 0.1% formic acid in acetonitrile, B = 0.1% formic acid in water, and Flow Rate = 0.6 mL/min, Gradient. MS Settings used were as follows: Mode of Operation = Tof MSE, Ionization = ESI (−)/ESI (+), and Acquisition Range = 50–1200 Da.

Screening for active compounds in natural products is carried out using LCMS/MS-QTOF with UNIFI software (version 1.6), which contains a library of mass spectra of active compounds from the Waters database. The UNIFI software (version 1.6) allows the identification of mass spectra of compounds in the sample, which are then matched with the mass spectra in the library.

### 2.3. In Silico Molecular Docking Analysis

The research was conducted on an ASUS Vivobook M413ia—Ek502t laptop with a 2.3 GHz AMD Ryzen 5 4500u processor, 8 GB DDR4 memory, and 512 GB SSD M.2 storage. This laptop was equipped with ChemDraw Ultra 12.0, AutoDock tools (version 4.2), and BIOVIA Discovery software (version 20.1), as well as the Windows 10 Home operating system and the AutoDock tools (version 4.2). In addition, the researchers utilized the Protein Data Bank and PubChem structure databases. The study′s protocols for molecular coupling simulation were based on previously established methodologies [20].

From the EBN profiles, specific compounds were identified for the test ligands. These compounds were created in 2D with ChemDraw Ultra 12.0, converted to 3D, and optimized using the MM2 algorithm. The focus of the investigation was on four Protein Data Bank-obtained target proteins: human inducible nitric oxide synthase (PDB ID: 3E7G), human reactive oxygen species 1 kinase (PDB ID: 3ZBF), human pancreatic lipase (PDB ID: 1LPB), and fat mass and obesity-associated protein (PDB ID: 3LFM).

To validate the process of molecular docking, redocking was performed. Using precise grid coordinates, AutoDock tools (version 4.2) were used to position the original ligand back into the target binding site. After redocking, the ligand′s position was evaluated, and it was required to have a root-mean-square deviation (RMSD) of less than 2.0. Grid and docking parameter adjustments were made based on the results of the docking validation. The final conformation structure of each docking was stored as a *dlg file, and Discovery Studio 2016 was used to analyze the interaction between the ligands and receptors.

### 2.4. Antioxidant Activity by ABTS and DPPH Radical Scavenging Activity Assays

Following the methodology described in prior investigations [21,22], the scavenging activity of 2,2′-azino-bis(3-ethylbenzothiazoline-6-sulfonic acid) (ABTS+; Sigma-Aldrich, Saint Louis, MO, USA) was determined. To produce the ABTS working solution, 7 mM ABTS, and 2.4 mM potassium persulfate were combined in equal proportions and incubated for 14 h at 22 °C in a dark container. The mixture was then diluted to produce a new working solution with an absorbance of 0.706% at 734 nm. Various concentrations of EBN preparations (50, 100, 150, 200, and 250 μg/mL) were diluted with 1 mL of the ABTS working solution for the ABTS scavenging assay. After a 7-min incubation period, each sample′s absorbance at 734 nm was measured. In this investigation, the positive control was Trolox. The procedures detailed in previous investigations were followed [18,21,22]. 

The 2,2-diphenyl-1-picrylhydrazyl radical-scavenging activity (DPPH) test was performed. Different concentrations of EBN preparations (50, 100, 150, 200, and 250 µg/mL) were added to containers containing 3 mL of DPPH reagent. After 30 min of incubation at ambient temperature, the absorbance of each sample was measured at 517 nm. This assay utilized glutathione (GSH; Sigma-Aldrich, 354102) as the positive control. To assure accurate and reliable results, each sample was subjected to triplicate analysis (*n* = 3) for both the ABTS and DPPH assays. As determined by the experimental procedure, the inhibition of DPPH and ABTS was calculated using the corresponding formula. Inhibition of DPPH and ABTS was determined according to the following formula:

$$Inhibition\;Activity\;\left(\%\right)=\frac{A0-A1}{A0}\times 100\%$$

where *A*0 = absorbance of blank; and *A*1 = absorbance of standard or sample.

The half-maximal effective concentration ratio (EC_50_) with Trolox for the ABTS test and glutathione for the DPPH assay, the radical-scavenging capacity of EBN (Edible Birds Nest), was expressed. EC_50_ stands for the sample concentration at which the initial radical concentration is reduced by 50%. In other words, the sample′s capacity to scavenge free radicals is represented by the EC_50_ value, with lower values indicating greater antioxidant activity. To compare the radical-scavenging capacities of EBN against well-known antioxidants, tronx and glutathione were employed as reference molecules.

### 2.5. In Vitro Antidiabetic Assay via α-Glucosidase and α-Amylase Inhibition

The researchers performed two different inhibitory activity tests on the EBN samples, as per methodologies described in previous literature [23,24]. The α-amylase inhibition activity of EBN samples was measured based on the previous literature [18,25]. For the α-glucosidase inhibition test, a phosphate buffer solution with a volume of 50 mL (pH 6.9) containing the enzyme was prepared. The enzyme concentration in the solution was 1.52 UI/mL. Maltose and sucrose solutions were added to the mixture, followed by the addition of EBN samples at various concentrations (ranging from 50 µg/mL to 250 µg/mL). Each sample was then mixed and incubated at 37 °C for 20 min. The enzyme was later inactivated by heating the tubes at 100 °C for 2 min. Acarbose was used as a positive control in this experiment. Regarding the α-amylase inhibition activity, diluted EBN samples were incubated at five different concentrations (ranging from 50 µg/mL to 250 µg/mL) along with NaCl (0.006 M), sodium phosphate buffer (pH 6.9), and porcine pancreatic amylase (0.5 mg/mL). Afterward, each mixture was combined with 500 µL of 1% starch solution and incubated at 25 °C for 10 min. Following this, 3,5-dinitro salicylic acid was added to complete the reaction, and the mixture was incubated at 100 °C for 5 min. After cooling at 22 °C, the absorbance of each sample was measured at 540 nm after dilution with distilled water. Acarbose served as the positive control in this case as well.

### 2.6. In Vitro Antiobesity Evaluation via Lipase Inhibition Assay

The inhibitory data was obtained using the previously described equation [23,24]. Initially, crude pig pancreatic lipase (PPL, 1 mg/mL) was dissolved in a 50 mM phosphate buffer (pH 7) prior to centrifugation at 12,000 g to remove insoluble components. In order to create an enzyme stock (0.1 mg/mL), the supernatant was diluted 10 times with a buffer. Based on prior research [26], a 96-well microplate containing 100 µL of EBN samples was combined with 20 L of 10 mM p-nitrophenyl butyrate (pNPB) in a buffer and incubated at 37 degrees Celsius (C) for 10 min. Orlistat (C29H53NO5, PubChem CID: 3034010), a well-known PPL or lipase inhibitor, was used as a comparison drug. At 405 nm, measurements were taken using a DR-200Bc ELISA microplate reader. Using the yield of the reaction rate of 1 mol of p-nitrophenol (4-nitrophenol, C6H5NO3) per minute at 37 degrees Celsius (C), the unit of activity was calculated. To determine the lipase inhibition activity, the PPL activity in the test mixture was decreased by a predetermined amount. To assure the validity of the study′s findings, each sample was verified three times (*n* = 3) or in triplicate.

$$Inhibition\;of\;Lipase\;Activity\;\left(\%\right)=100-\frac{B-Bc}{A-Ac}\times 100\%$$

where *A* = Activity without inhibitor; *B* = Activity with inhibitor; *Ac* = Negative control (−) without inhibitor; *Bc* = Negative control (−) with inhibitor.

### 2.7. In Vivo Study Design on Rats Fed on Cholesterol- and Fat-Enriched Diet

#### 2.7.1. Animal Handling and Ethical Approval

Forty male Rattus norvegicus (*Rattus norvegicus*) rats weighing 204.03 ± 3.68 g were used in the in vivo investigation. The rats came from the Animal Model Farm in Yogyakarta, Indonesia, and were acclimated for 10 days in a controlled environment (27 °C, 50–60% relative humidity, balanced light–dark cycle), with access to regular animal feed and water from PT Citra Ina Feedmill. The study protocol gained ethical approval from the International Register of Preclinical Trial Animal Studies Protocols (preclinicaltrials.eu; https://preclinicaltrials.eu, accessed on 22 March 2023) with registration number PCTE0000371 and complied with the Guidelines for Reporting In Vivo Experiments (ARRIVE).

#### 2.7.2. Study Design of Treatments

The rats were randomly separated into four treatment groups following the acclimation phase. Groups C and D were also fed CFED diets and water ad libitum but with daily supplements of 22.5 (low dose/LD) and 45 (high dose/HD) mg/kg body weight (BW) of EBN, respectively. Group A received a normal diet and water ad libitum. Group B was fed a cholesterol- and fat-enriched (CFED) diet with ad libitum water. The oral EBN doses were given by a professional and administered by oral gavage. Throughout the study, the daily intake of food and water was observed, and there were no changes between the control and experimental groups in this respect.

#### 2.7.3. Feed or Pellet Composition and CFED Production

The typical pellets are purchased from Rat Bio^®^ in Jakarta, Indonesia, and have the following composition percentages: 12% moisture, 20% protein, 4% fat, 14% calcium, 1% fiber, 0.7% phosphorus, 11.5% total ash, 0.3% vitamin C, and 0.1% vitamin E. According to the recommendations of the manufacturer, these pellets were properly stored in a cool, dry location away from direct sunlight.

A technique based on earlier research [26,27] was used to develop the CFED diet. The procedure involves adding certain additives to the dry, standard pellets. These additions comprised 2% powdered cholesterol, 2% maize oil, 20% animal fat, and 1% cholic acid. After homogenizing the mixture, 1 L of distilled water was used to break the mixture pellets into smaller pieces. The pellets were initially dried sterile at ambient temperature before being kept at 4 °C to reduce oxidation. The CFED diet is composed of the following: carbs make up 43.6% of the diet, protein makes up 12.4%, fiber makes up 4.7%, fat makes up 3.2%, cholesterol makes up 2%, cholic acid makes up 1%, animal fat makes up 20%, total ash makes up 4%, maize oil makes up 2%, and the rest is moisture.

#### 2.7.4. Biomedical Analysis of Collected Blood Samples

Blood samples were taken six weeks after the rats received interventional feeding. The animals were fasted overnight and given ketamine as anesthesia prior to the blood collection. Blood was extracted from the venous sinus and put in an unanticoagulated, dry, and sterile tube. The blood was then left to coagulate at room temperature. The serum was collected after 20 min of centrifugation at 3000 rpm. The COBAS Integra^®^ 400 Plus Analyzer (Roche Diagnostics, Basel, Switzerland) was used to conduct biomedical studies of several parameters. Low-density lipoprotein (LDL), triglycerides (TG), high-density lipoprotein (HDL), total cholesterol (TC), and blood glucose (BG) levels were among them. Blood was also drawn from the cardiac tissue in order to evaluate other biomarkers. Using the SOD Assay Kit from Sigma-Aldrich, the superoxide dismutase (SOD) enzyme activity was assessed. The Mouse Pancreatic Lipase ELISA Kit (Merck KGaA, Darmstadt, Germany) was used to assess blood lipase levels, and the Mouse Pancreatic Amylase ELISA Kit to measure serum amylase levels. Using particular ELISA kits, the levels of the inflammatory biomarkers PGC-1 (peroxisome proliferator-activated receptor-gamma coactivator-1 alpha), TNF-alpha (tumor necrosis factor-alpha), and IL-10 (interleukin 10) were measured. PGC-1, TNF-alpha, and IL-10 were evaluated using kits from Sunlong Biotech Co., Ltd. (Zhejiang, China), PGC-1 Mouse ELISA Kit from Abcam, and Mouse Tumor Necrosis Factor-alpha (TNF-alpha) Kit from Sunlong Biotech Co., Ltd. (Zhejiang, China). Additionally, during the investigation, the body weights of the rats were determined using digital scales.

### 2.8. Data Analysis and Management

Numerous statistical techniques were used in the data analysis and management area to evaluate the experimental outcomes both in vitro and in vivo. The unpaired *t*-test was utilized to establish the statistical significance of the in vitro investigations, which included antioxidant inhibition of ABTS and DPPH, as well as inhibitory actions for lipase, -amylase, and -glucosidase. In order to ensure reproducibility, these tests were carried out in triplicate. Nonlinear regression methods were utilized to construct each EC_50_ dataset, which helped determine the sample concentration that resulted in a 50% reduction in the starting radical concentration. Several factors for in vivo data processing were investigated. Multivariate analysis of variance (ANOVA) was used to examine the lipid profile (LDL, HDL, TG, TC, and BG), inflammatory biomarkers (IL-10, TNF, and PGC-1), and enzymatic tests (SOD cardio, serum lipase, and serum amylase). This made it possible to look at several variables′ connections at once. Pairwise *t*-tests (or dependent t-tests) were used to determine whether there were any significant differences in body weights (g) within each group between the starting and finishing points. Additionally, a one-way ANOVA was used to find differences between each secondary parameter, including the beginning, final, and daily weight gain (g/day) for each group, water intake (mL), food intake (g), the food efficiency ratio (FER), and water consumption. With a 95% level of confidence, all reported data were given as mean values with a standard error of the mean (SEM). Data analysis on MacBook laptops was done using GraphPad Prism 9.4.1 software.

## 3. Results

### 3.1. Terpenoids Observed in EBN

The analysis of compounds in natural products is very important for the basic characterization of potential components in them. In this study, four terpenoid compounds (Bakuchiol, Curculigosaponin A, Dehydrolindestrenolide, and 1-methyl-3-(1-methyl-ethyl)-benzene) have been observed in EBN through LCMS/MS-QTOF analysis, and the data are presented in Table 1, which have met the criteria: Mass error ≤ 5 ppm; Isotope match MZ RMS PPM ≤ 6 ppm; Isotope match MZ RMS % ≤ 10%; Intensity/Response ≥ 300; and Fragment match ≥ 1 mass fragment. Chromatogram mass spectrum data can be viewed in Supplementary Materials File S1.

The four compounds observed in EBN (Table 1) were continued preparation for in silico molecular docking tests.

### 3.2. In Silico Molecular Docking Simulation in Selected Receptors

The observed metabolites, Bakuchiol, Curculigosaponin A, Dehydrolindestrenolide, and 1-methyl-3-(1-methyl-ethyl)-benzene were then followed by in silico or molecular docking studies on selected receptors for antioxidants and obesity. The molecular docking assay focuses on specific receptors such as human inducible nitric oxide synthase (iNOS), human pancreatic lipase, human reactive oxygen species (ROS) 1 kinase, and fat mass and obesity-associated (FTO) proteins. All these receptors were continued with molecular docking validation tests and obtained an assessment accuracy value of less than 2 Å which indicates they are successfully validated; the data are shown in Table 2.

After being successfully declared valid, molecular docking simulations were carried out between ligands or identified compounds from EBN against selected receptors, and molecular docking value data, or ΔG (kcal/mol), is presented in Table 3. Based on Table 3, molecular docking results show that the four terpenoids of EBN have higher ΔG values than standard control drugs (orlistat) in terms of antiobesity potential (potential inhibiting FTO protein and human lipase enzymes). Interestingly, Curculigosaponin A has inhibitory activity against three of the four selected receptors and has a higher value than standard drugs as controls, especially being more potent in inhibiting iNOS than S-ibuprofen. More interestingly, Bakuchiol and Dehydrolindestrenolide are considered very potential candidates for antioxidant and antiobesity-related metabolic syndrome because they have higher inhibitory power at all four receptors than standard drugs as controls, including the ROS1 kinase receptor. This proves molecularly that the compound components in EBN have the potential to have antioxidant and antiobesity properties in an in silico molecular docking simulation.

Visualization of the interaction of amino acids from the active compound EBN against iNOS, ROS1 kinase, FTO, and lipase can be seen in Supplementary File S2. Indeed, in silico studies above have observed the potential of EBN against selected receptors. However, further studies of in vitro biological activity are needed to validate these results. In vitro studies in this study have also been conducted and reported in the sections below.

### 3.3. In Vitro Study Reveals the Antioxidants, Antidiabetic, and Antiobesity Potential of EBN

**Figure 1:**
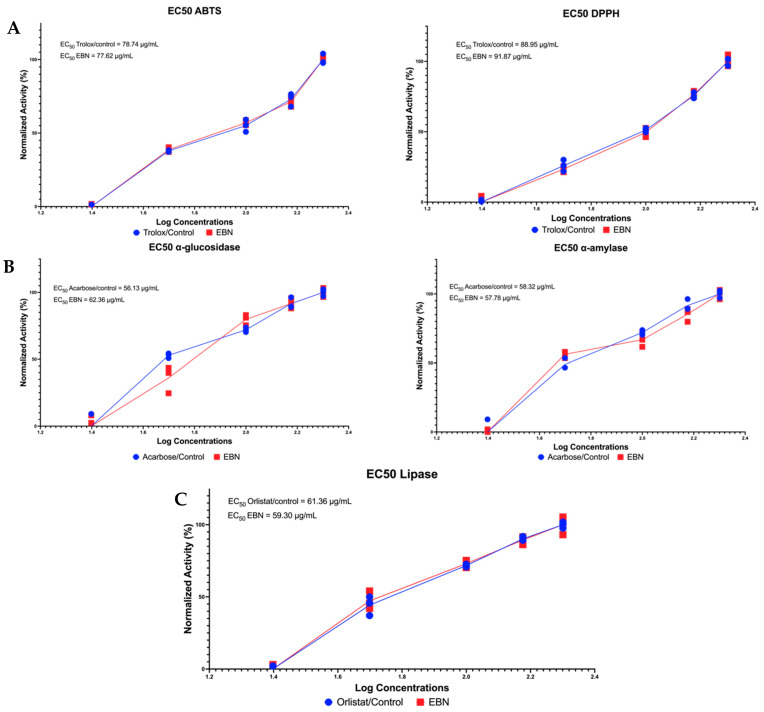
In vitro antioxidants, antidiabetic and antiobesity activities of EBN. (**A**) EC_50_ antioxidants via ABTS and DPPH inhibition activity. (**B**) EC_50_ antidiabetic via α-glucosidase and α-amylase inhibition activity. (**C**) EC_50_ antiobesity via lipase inhibition activity.

The results of the in vitro study presented in Figure 1 are in line with the results of in silico tests described in the previous section, indicating that EBN indeed has potential as a promising antioxidant, antidiabetic, and antiobesity compound. Judging from the EC_50_ value in the antioxidant activity test via ABTS inhibition activity with a value of 77.62 μg/mL, which is smaller than Trolox (78.74 μg/mL), a control, this shows that EBN is more potent in free radical scavenging than Trolox (EC_50_ EBN < EC_50_ Trolox). Furthermore, the EC_50_ value of EBN in α-amylase inhibition activity also shows similar things to the ABTS, EC_50_ EBN < EC_50_ Acarbose or control tests (Figure 1). Interestingly, the EC_50_ EBN in lipase inhibition activity has a value of 59.30 μg/mL, which is more potent in antiobesity candidates than orlistat or control, which has a greater EC_50_ value (61.36 μg/mL). Overall, these in vitro tests are consistent with in silico tests that reveal the health effects of EBN in terms of metabolic syndrome.

To provide comprehensive knowledge of the health benefits of EBN, especially as a functional food candidate that can fight metabolic syndrome, in vivo tests on animal models were also conducted and reported in this study.

### 3.4. In Vivo Study Reveals Attenuation Metabolic Syndrome by EBN Supplementation

Data characteristics of experimental rats (*R. norvegicus*), including body weight (BW), feed, water intake, and FER, are presented in Table 4. It is clear that the initial BW of all groups is the same, and there is no significant difference. The BW, food intake, and water intake of experimental rats were calculated daily and obtained values that did not differ significantly in each group during treatment. However, interestingly, it was clear that the CFED diet had significantly higher final BW values than both the normal and EBN treatment groups. Furthermore, the group fed the diet with a second dose of EBN had significantly lower final BW values than CFED and even the normal group (*p* < 0.05). The group with a high dose of EBN supplementation had the lowest final BW score of the other groups.

**Figure 2:**
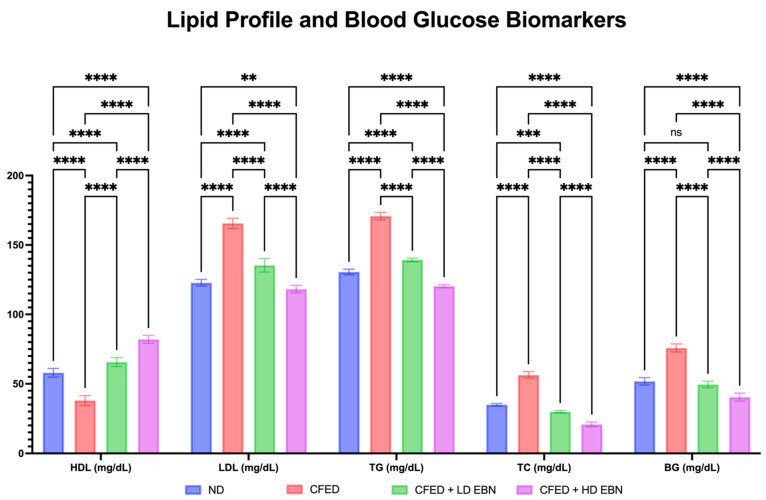
Improvements in lipid profile and blood glucose were observed in experimental rats. **** *p* < 0.0001, *** *p* = 0.0004, ** *p* = 0.0011, and ^ns^ *p* > 0.05.

Improvements in lipid and blood glucose profiles were also observed in the CFED group given EBN supplementation at both doses; the data are presented in Figure 2. In general, high LDL, TG, TC, and BG values and low HDL levels were observed significantly in the CFED group when compared to the normal control group. Both doses of EBN were significantly observed to upregulate levels of HDL and downregulate levels of LDL, TG, TC, and BG. Interestingly, high-dose EBN supplementation was significantly better at reducing levels of LDL, TG, TC, and BG and HDL enhancement when compared to normal and low-dose EBN controls. Overall, high-dose EBN supplementation led to the modulation of both lipid profiles and blood glucose in CFED rats. This is possible because there are two terpenoids, namely Bakuchiol and Dehydrolindestrenolide, which in in silico molecular docking can inhibit lipase and FTO protein (Table 3).

**Figure 3:**
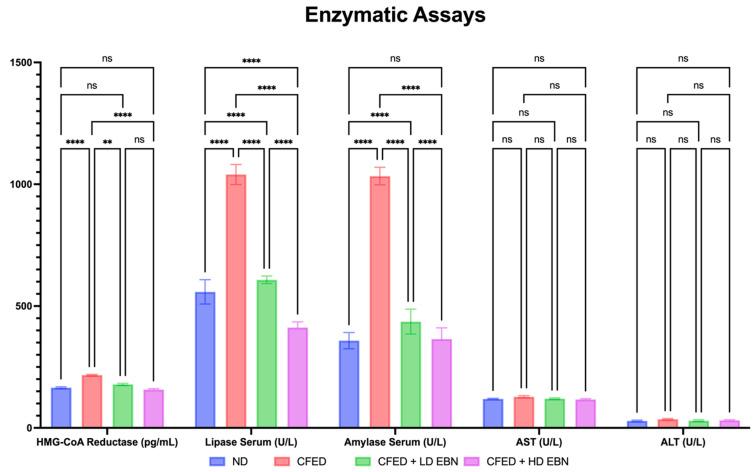
Improvements in metabolic syndrome-related enzymatic levels observed in experimental rats. **** *p* < 0.0001, ** *p* = 0.0036, and ^ns^ *p* > 0.05.

In addition to improvements in lipid profiles and blood glucose (Figure 2), improvements in the metabolic enzymes HMG-CoA reductase, lipase, and α-amylase were observed in experimental rats supplemented with EBN, and the data can be seen in Figure 3. In general, CFED-only rats had significantly higher levels of HMG-CoA reductase, lipase, and α-amylase when compared to both the normal and EBN intervention groups. Again, it was found that HD of EBN led to improvements in the metabolic enzymes HMG-CoA reductase, lipase, and α-amylase, although both doses of EBN also had a good effect on decreasing these enzymes. Interestingly, serum aspartate transaminase (AST) and alanine transaminase (ALT) were observed within normal limits, and there were no significant differences across treatment groups. This can be a marker of less toxicity for EBN as a candidate for functional food that is already widely consumed by humans.

**Figure 4:**
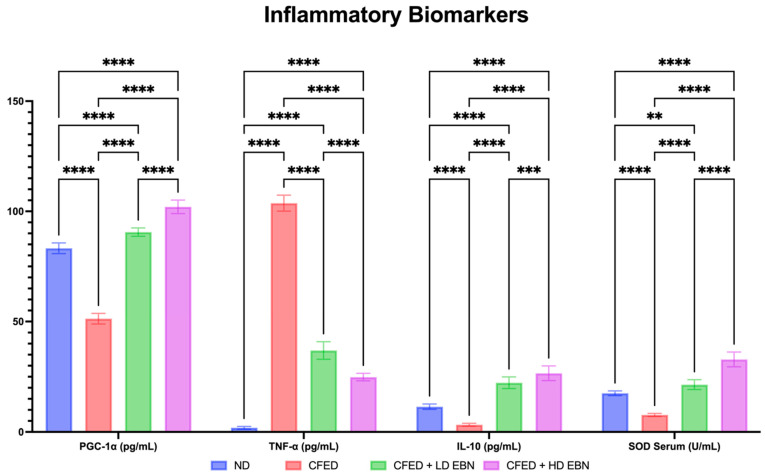
Improvements of metabolic syndrome-related inflammatory biomarkers observed in experimental rats. **** *p* < 0.0001, *** *p* = 0.0008, and ** *p* = 0.0024.

CFED rats supplemented with both doses of EBN had significant improvements in inflammatory biomarkers, including peroxisome proliferator-activated receptor-γ coactivator 1-α Levels (PGC-1α), tumor necrosis factor-alpha (TNF-α), interleukin 10 (IL-10), and superoxide dismutase (SOD) serum levels and can be seen in Figure 4. In addition, it generally appears that the CFED group did have high levels of TNF-α and levels of PGC-1α, IL-10, and SOD that were significantly lower than the normal diet group and the EBN intervention group. HD of EBN continues to lead the way in upregulating levels of PGC-1α, IL-10, and SOD and downregulating TNF-α. This comprehensively shows that EBN, in addition to improving lipid profiles, blood glucose, and metabolic enzymes, can also help modulate inflammatory biomarkers, making it a functional food candidate that also has the potential as an anti-inflammatory related to metabolic syndrome.

## 4. Discussion

Edible bird nest (EBN) is consumed in many parts of the world and has been developed into several processed foods; some markets even claim that it is healthy for the body. However, unfortunately, these claims are still poorly supported by evidence-based medicine. In particular, studies of the characterization of metabolite components such as terpenoids and in silico molecular docking analysis of receptors that cause non-communicable diseases such as metabolic syndromes are limited. Furthermore, in vitro and animal model studies regarding the supplementation of EBN are urgently needed to complement the current evidence of its health benefits, especially in fighting metabolic syndrome, and this is the urgency of current research. 

**Figure 5:**
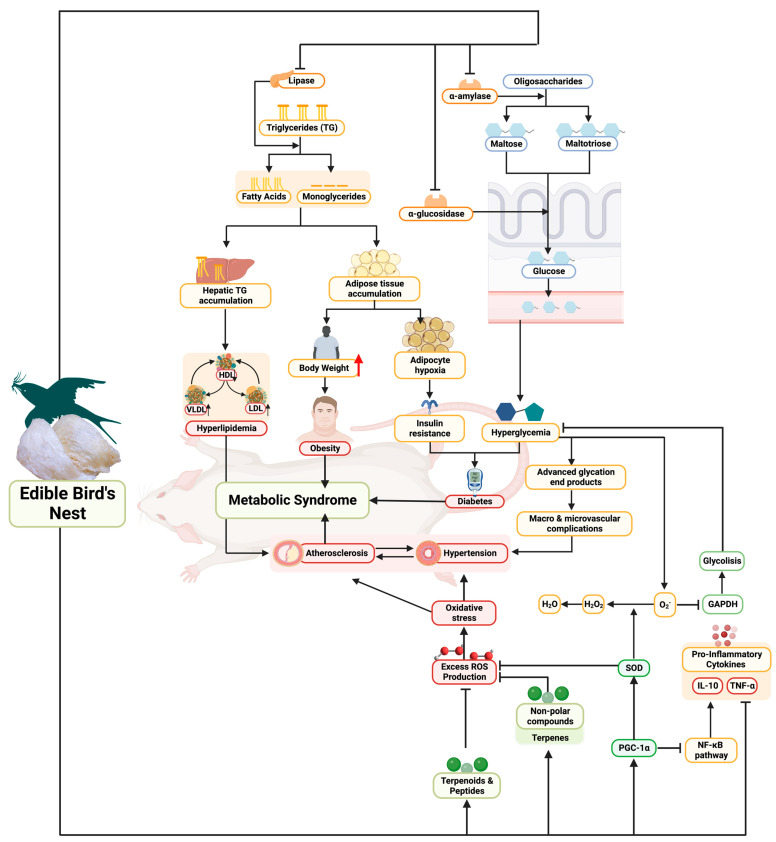
Biomechanism of EBN as novel functional foods in combating metabolic syndrome.

Metabolic syndrome is closely related to the incidence of oxidative stress and several related diseases such as obesity and diabetes (Figure 5) [28]. In people with metabolic syndrome, iNOS levels are found to be higher, and this correlates with the incidence of oxidative stress [29]. In addition, lowering and inhibiting ROS1 kinase, FTO protein, and lipase can reduce the incidence of metabolic syndrome in obese people, and inhibiting these receptors can also be an anti-aging approach [20,30,31]. In this current study, four kinds of terpenoid compounds in EBN have been identified, and molecular docking simulations show activity in inhibiting iNOS, ROS1 kinase, FTO protein, and lipase, which has never been reported in similar studies before. Even two of the four compounds observed clearly showed the potential of EBN in inhibiting iNOS receptors, ROS1 kinase, FTO protein, and lipase higher than the control drugs, namely Bakuchiol and Dehydrolindestrenolide. Bakuchiol has been shown to have pharmacological effects, including as an antioxidant, reducing blood glucose and triglycerides, and anti-inflammatory effects [32]. Interestingly, our study complements previous findings that have explored Dehydrolindestrenolide as a new cancer-fighting sesquiterpene [33]. EBN is more complete with the content of Curculigosaponin A, which has been shown by some studies to be a therapeutic alternative in obesity-related metabolic syndrome [34]. Interestingly, the results of this study can provide insight into the pharmaceutical industry to develop and synthesize the observed Bakuchiol and Dehydrolindestrenolide from EBN to be used as further treatments for metabolic syndrome. A systematic review has further analyzed the nutritional bioactive compounds of EBN, but only focused on bioactive glycopeptides [35]. In addition, the latest study also recently focused on protein profiling of EBN [36]. Therefore, most recent EBN studies have focused on protein profiles and included bioactive peptides. Our study presents the latest findings that provide information on the content of terpenoids in EBN and their molecular activity computationally or in in silico. 

In in vitro studies, the EBN antioxidants in our study complement previous studies. Oxidative stress, which is the result of an imbalance in the homeostasis of the reduction-oxidation reaction between prooxidants and antioxidants, is a major player in the pathogenesis of many diseases such as metabolic, inflammatory, degenerative, and cardio-vascular (Figure 5) [37]. To counteract these pathological mechanisms, exogenous antioxidants are needed that will act interactively and synergistically with endogenous antioxidant defense systems to maintain the homeostasis of reduction-oxidation (redox) reactions [37]. A meta-analysis and systematic review study of randomized clinical controls showed that antioxidant supplementation is essential for the improvement of metabolic disorders in obese patients [38]. The role of pancreatic lipase inhibitors in lipid metabolism is very important in reducing hyperlipidemia, especially in obese patients with metabolic syndrome [39]. Lipase inhibitors from natural ingredients are very important to explore further, like this EBN, which has a higher ability to inhibit lipase than control or orlistat drugs (Figure 1). People with obesity relatively have a higher risk of developing type 2 diabetes [40], and α-amylase inhibitors from natural ingredients are an alternative therapy. In addition, in vitro antidiabetic EBN showed higher potential in inhibiting α-amylase compared to acarbose as a drug or control (Figure 1). Our study complements previous findings showing that EBN has been shown to fight hyperglycemia and oxidative stress [41]. 

In metabolic syndrome, which is also characterized by inflammation, PGC-1α dysregulation modifies the metabolic properties of tissues by altering mitochondrial function and inducing ROS accumulation (Figure 5) [42]. Hence the importance of maintaining PGC-1α levels and increasing them, for balance and control of mitochondrial DNA replication and cellular oxidative metabolism [43]. Rats supplemented with high doses of EBN were found to have higher levels of PGC-1α than other groups, and this upregulation is an important key role of EBN in modulating metabolic syndrome. In addition, it is important to find new HMG-CoA reductase inhibitors as new alternatives to fight metabolic syndrome because HMG-CoA reductase inhibitors can reduce TC, LDL, and TG concentrations and increase HDL concentrations with various potentials [44,45]. EBN has been clearly significant in modulating or downregulating metabolic enzymes such as HMG-CoA reductase, lipase, and α-amylase serum, decreased blood glucose suppression, lipid profile, and weight loss in EBN-treated rats. The decrease in LDL, TG, TC, and BG is strongly suspected to contribute to the reduction of fat stores and free fatty acids in many organs and/or tissues, which can be seen from the resulting weight loss [46]. Metabolic syndrome is also characterized by an increase in oxidative stress which contributes to impaired inflammation [47]; thankfully, EBN in silico, in vitro, and in vivo can be anti-inflammatory. In this in vivo study, HD of EBN continues to lead in upregulating levels of PGC-1α, IL-10, SOD, and downregulation of TNF-α; which will reduce the complications of metabolic syndrome (Figure 5). Several studies of other natural ingredients also support the potential for improvement of metabolic syndrome, such as green algae (*Caulerpa racemosa*) extract [18,24,27] and *Clitoria ternatea* Kombucha [20,22], which have an improving effect on lipid profiles and inflammation. However, if we compare *C. racemosa* extract and *C. ternatea* with EBN, EBN is the one that is easier to consume and prepare (considering that there are already many on the market).

This study managed to comprehensively uncover the remedial effects of fighting metabolic syndrome through integrated studies in silico, in vitro, and in vivo, which have never been reported before. This study is the first study to successfully identify terpenoid components in EBN and analyze their molecular activity against selected receptors for metabolic syndrome computationally in silico. This in vivo study was also the first to successfully explore the modulation of PGC-1α and HMG-CoA reductase by EBN. However, this research is only at the stage of animal models, which certainly cannot represent the results in human clinical trials. Therefore, further human clinical trials with doses derived from this reported in vivo study are needed. In addition, limited funding has resulted in researchers not being able to conduct fecal microbiota analyses that other studies are expected to do in the future to complement current knowledge about EBN. Furthermore, EBN is a complex mixture of various components that may vary depending on the origin, processing, and storage of the product; therefore, further research focusing on the basic characterization of EBN from all islands in Indonesia complements current knowledge.

## 5. Conclusions

Something new and comprehensive related to the health benefits of edible bird nests (EBN) has been reported in this study, especially in the fight against metabolic syndrome. A total of four terpenoids contained in EBN were successfully identified, and two of the four compounds (Bakuchiol and Dehydrolindestrenolide) had great activity in inhibiting iNOS, ROS1 kinase, FTO, and lipase via in silico molecular docking simulation. Interestingly, in vitro studies validated the observed effects of in silico studies and provided insight into antioxidants, antidiabetic, and antiobesity of EBN, which is certainly associated with metabolic syndrome. More interestingly, in vivo tests revealed that improvements in lipid profiles, blood glucose, inflammatory biomarkers, and also enzymatic levels were obtained in rats supplied by EBN with high doses. Finally, this study has revealed the potential of EBN as a new functional food candidate against metabolic syndrome, and of course, clinical trials in humans are needed to see further efficacy.

## 6. Patents

The preparation method and formulation of edible bird nest as novel functional foods in combating metabolic syndrome resulting from the work reported in this study have been registered as a patent in Indonesia (Fahrul Nurkolis is the patent holder of the EBN Formulation).

## Figures and Tables

**Table 1:** Confirmed terpenoids component in EBN via LCMS/MS-QTOF analysis.

Component Name	Formula	Observed RT (min)	Mass Error (ppm)	Total Fragments Found	Isotope Match Mz RMS PPM	Isotope Match Intensity y RMS Percent	Response	Adducts
Bakuchiol	C_18_H_24_O	16.82	0.7	2	0.90	2.93	817	+H
Curculigosaponin A	C_36_H_60_O_9_	17.19	−3.9	19	4.47	2.13	26866	+H
Dehydrolindestrenolide	C_15_H_16_O_2_	16.67	−1.4	17	3.32	3.19	1272	+H
1-Methyl-3-(1-methyl-ethyl)-benzene	C_11_H_16_	16.72	−1.4	12	1.58	2.41	1493	+H

**Table 2:** Validation of molecular docking simulation.

No.	Drug Target	PDBID	Docking Site (x;y;z)	Docking Area (x;y;z)	RMSD (Å)	ΔG (kcal/mol)	Numb in Cluster (/100)	Judgment (<2 Å)
1	iNOS	3E7G	55.022, 21.817, 78.677,	40 × 40 × 40	1.789	−6.67	98	Valid
2	ROS1 kinase	3ZBF	42.521, 19.649, 3.987,	40 × 40 × 40	1.216	−7.83	90	Valid
3	Human pancreatic lipase	1LPB	−0.423, 16.723, 26.546,	42 × 40 × 40	1.499	−4.13	26	Valid
4	Fat mass and obesity-associated (FTO) protein	3LFM	29.043, −6.644, −29.329,	42 × 42 × 42	0.715	−6.29	90	Valid

Protein data bank–PDB, Root mean square deviation–RMSD.

**Table 3:** Molecular docking parameter of identified terpenoid compounds of two EBNs.

No.	Substance	Number in Cluster (/100)				ΔG (kcal/mol)				Ki			
		3E7G	3ZBF	1LPB	3LFM	3E7G	3ZBF	1LPB	3LFM	3E7G	3ZBF	1LPB	3LFM
Control													
1	S-ibuprofen	33				−4.73				128.28 μM			
2	Trolox		100				−5.36				85.58 uM		
3	Orlistat			6	5			−2.38	−3.71			5.22 mM	212.83 uM
1	Bakuchiol	74	66	68	89	−5.85	−6.12	−5.94	−6.80	19.90 uM	9.04 uM	11.59 uM	6.14 uM
2	Curculigosaponin A	52	20	23	34	−5.53	−4.70	−6.51	−5.36	15.85 uM	26.89 uM	386.01 nM	10.51 uM
3	Dehydrolindestrenolide	100	100	100	100	−7.34	−6.95	−6.74	−7.04	4.14 uM	7.85 uM	11.38 uM	6.85 uM
4	1-methyl-3-(1-methyl-ethyl)-benzene	100	100	96	77	−4.47	−4.74	−4.47	−4.75	515.43 uM	329.80 uM	525.40 uM	268.68 uM

**Table 4:** Body weight, feed, water intake, and FER characteristics of experimental rats (*R. norvegicus*).

Group	Normal	CFED	CFED + Low Dose EBN	CFED + High Dose EBN	p **
Initial BW (g)	203.62 ± 2.99	203.11 ± 2.80	204.19 ± 4.32	205.20 ± 4.53	0.2805
Final BW (g)	244.63 ± 3.19	277.09 ± 6.70	235.48 ± 3.23	230.10 ± 2.01	<0.0001
*p* *	*<0.000001*	*<0.000001*	*<0.000001*	*<0.000001*	
Weight gain (g/day)	0.89 ± 0.08	1.61 ± 0.17	0.68 ± 0.08	0.55 ± 0.10	0.8004
Food intake (g)	4.79 ± 0.47	4.89 ± 0.50	4.94 ± 0.69	5.05 ± 0.87	0.9960
Water intake (mL)	5.44 ± 0.63	5.37 ± 0.83	5.26 ± 0.79	5.01 ± 0.61	0.9822
FER (%)	18.74 ± 2.09	33.01 ± 3.15	13.96 ± 2.22	11.39 ± 3.84	0.0004

* Dependent or paired *t*-test CI 95% (0.05). ** ANOVA CI 95% (0.05). Food Efficiency Ratio (FER) was calculated by dividing body weight (BW) gain by food intake.

## Data Availability

The data datasets generated and/or analyzed in this study are available on request from the corresponding author.

## Supplementary Materials

The following supporting information can be downloaded at: https://www.mdpi.com/article/10.3390/nu15183886/s1, Supplementary File S1: Chromatogram data from observed compounds in EBN; Supplementary File S2: Visualization of the amino acid interactions of the active compound EBN on iNOS, ROS1 kinase, FTO, and lipase.
