# Supplementary material for: Revealing Edible Bird Nest as Novel Functional Foods in Combating Metabolic Syndrome: Comprehensive In Silico, In Vitro, and In Vivo Studies
Source: Nutrients. 2023 Sep 6;15(18):3886. doi: 10.3390/nu15183886 (PMC10535673; doi:10.3390/nu15183886)

The BPI Plot of Sampel

Item name: 230526_Biotin 1 ppm_Pos
Channel name: 1: TOF MSᴱ BPI (50-1200) 6eV ESI+ : Integrated : Smoothed

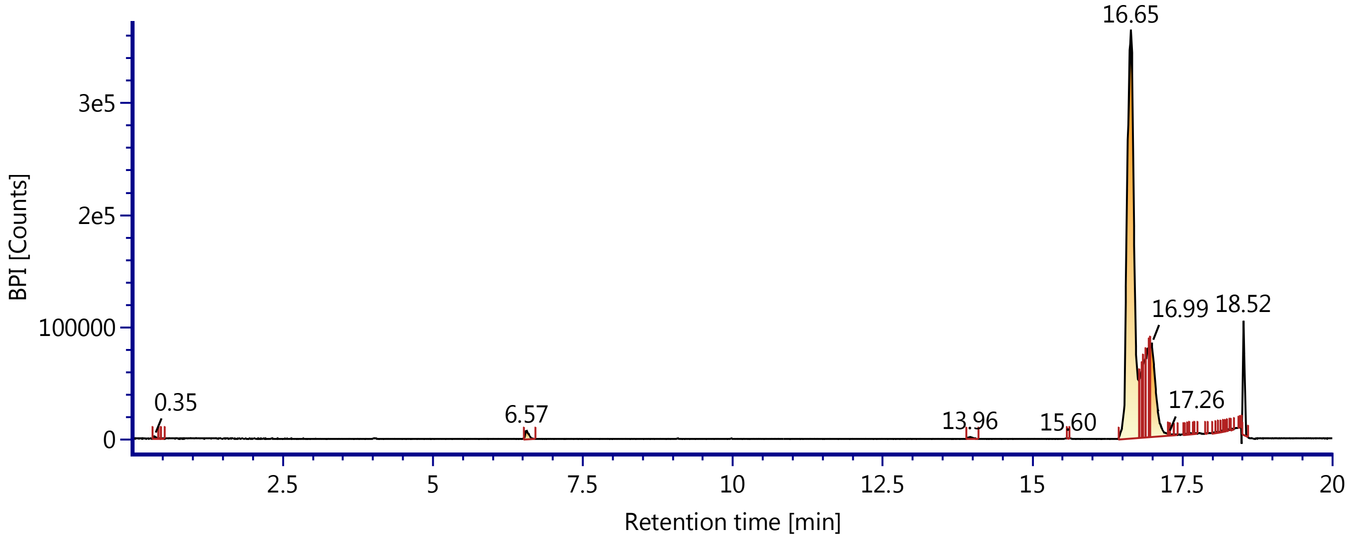

Component Plot

Item name: 230526_Biotin 1 ppm_Pos

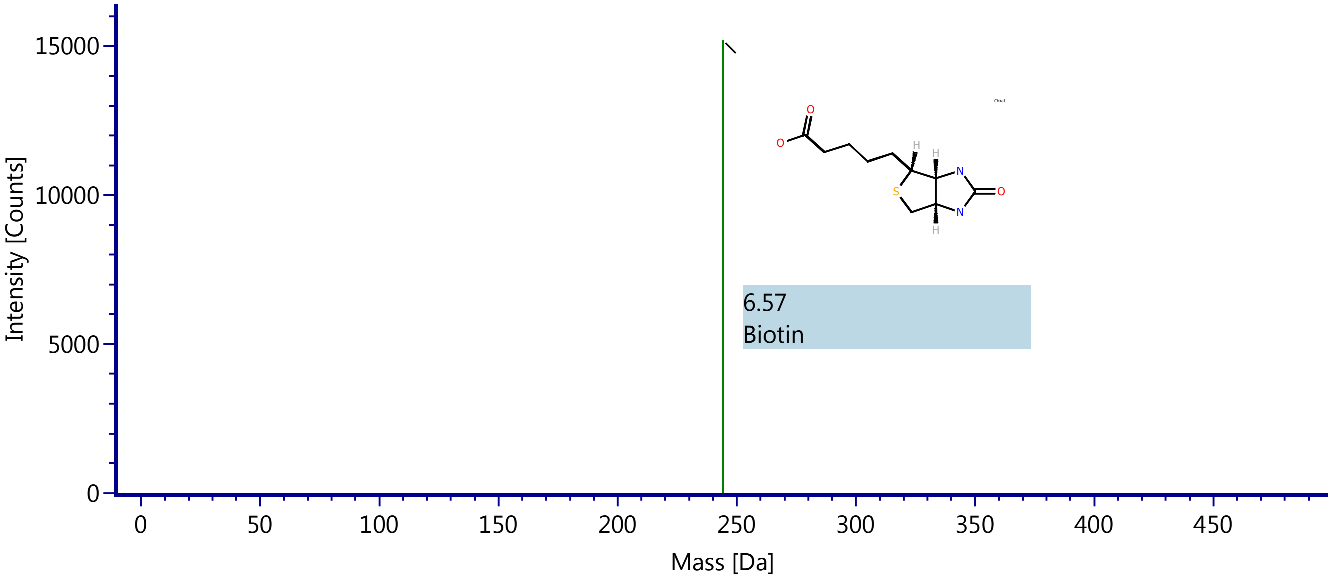

Component Summary (Confirmed)

Confirmed Component

Component name	Formula	Identification status	Observed RT (min)	Mass error (ppm)	Total Fragments Found	Isotope Match Mz RMS PPM	Isotope Match Intensity RMS Percent	Response	Adducts
Biotin	C10H16N2O3S	Identified	6.57	-0.4	11	1.42	0.63	15180	+H

Item name: 230526_Biotin 1 ppm_Pos
Channel name: Biotin [+H] : (33.3 PPM) 245.0954

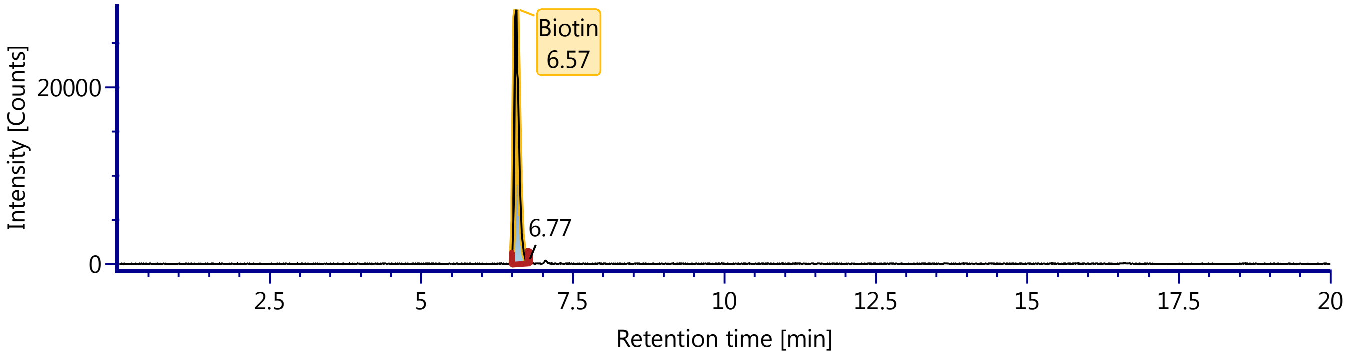

Item name: 230526_Biotin 1 ppm_Pos **Component name: Biotin**
Item description:

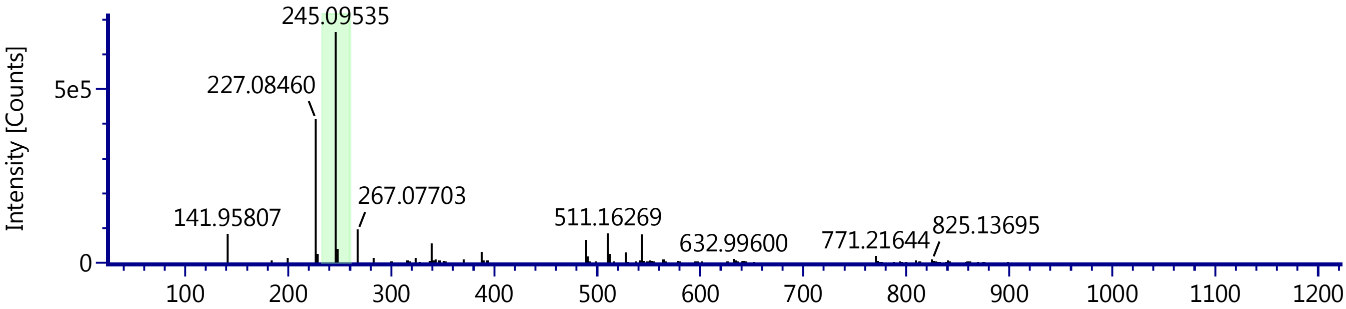

Item name: 230526_Biotin 1 ppm_Pos **Component name: Biotin**
Item description:

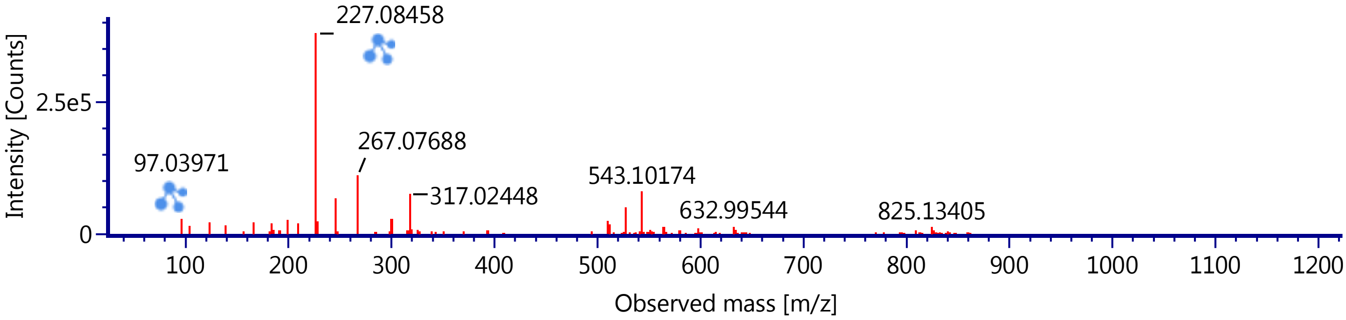

The BPI Plot of Sampel

Item name: 230526_MeOH_1_Pos
Channel name: 1: TOF MSᴱ BPI (50-1200) 6eV ESI+ : Integrated : Smoothed

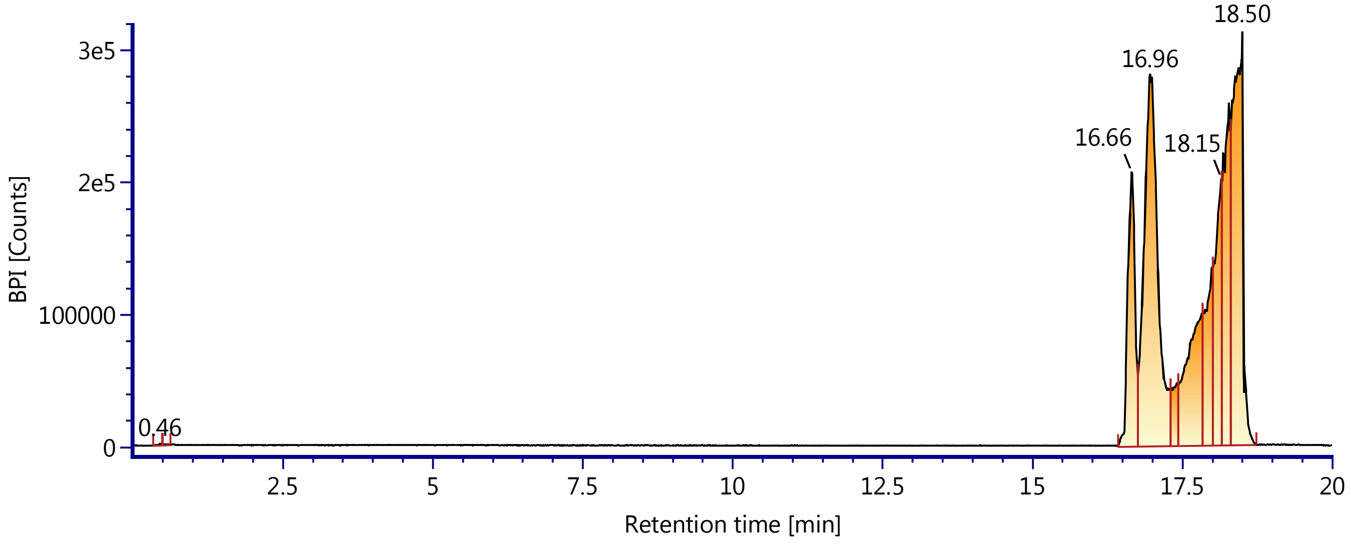

The BPI Plot of Sampel

Item name: 230526_305 R 93_1_Pos
Channel name: 1: TOF MS^E BPI (50-1200) 6eV ESI+ : Integrated : Smoothed

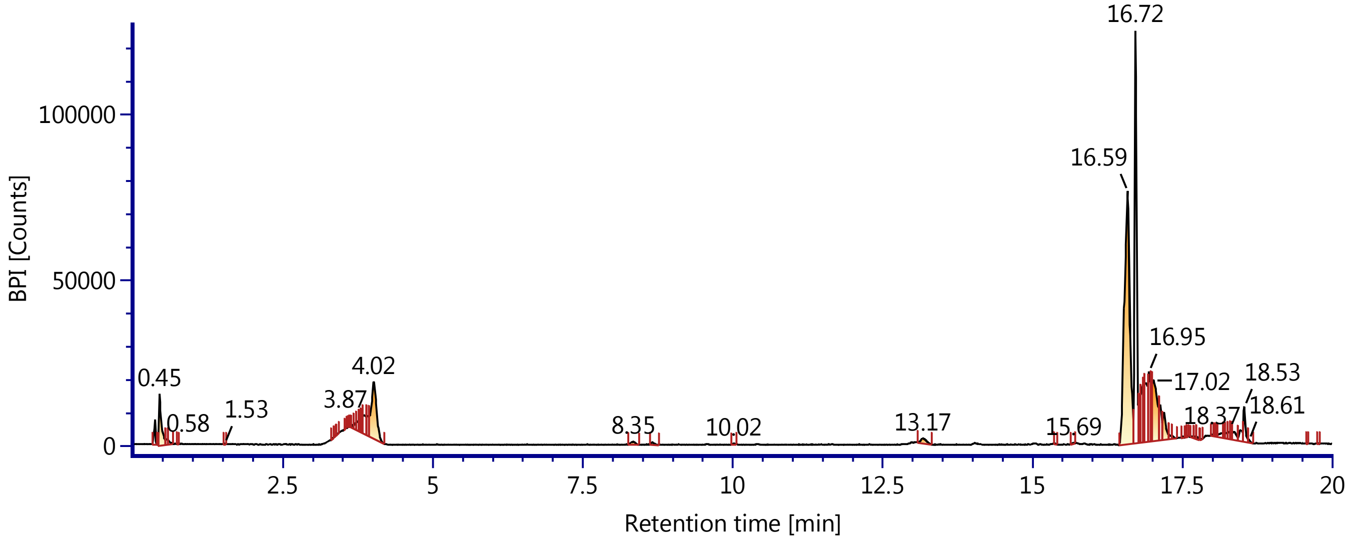

Component Plot

Item name: 230526_305 R 93_1_Pos

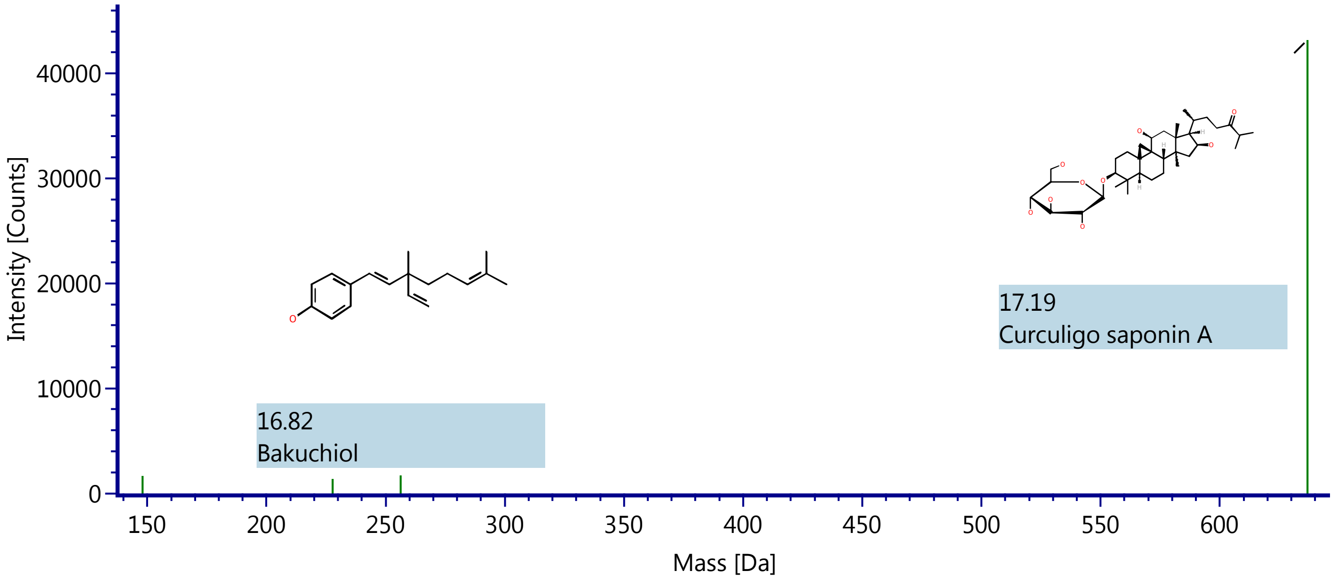

Component Summary (Confirmed)

Confirmed Component

Component name	Formula	Identification status	Observed RT (min)	Mass error (ppm)	Total Fragments Found	Isotope Match Mz RMS PPM	Isotope Match Intensity RMS Percent	Response	Adducts
1-Methyl-3-(1-methyl-ethyl)-benzene	$C_{11}H_{16}$	Identified	16.72	-0.8	10	0.98	3.57	1606	+H
Bakuchiol	$C_{18}H_{24}O$	Identified	16.82	-5.5	6	5.06	7.58	1681	+H
Curculigo saponin A	$C_{36}H_{60}O_9$	Identified	17.19	-3.9	38	4.31	2.61	43121	+H
Dehydrolindestrenolide	$C_{15}H_{16}O_2$	Identified	16.67	-2.7	25	2.52	3.81	1339	+H

Item name: 230526_305 R 93_1_Pos
Channel name: 1-Methyl-3-(1-methyl-ethyl)-benzene [+H] : (33.3 PPM) 149.1324

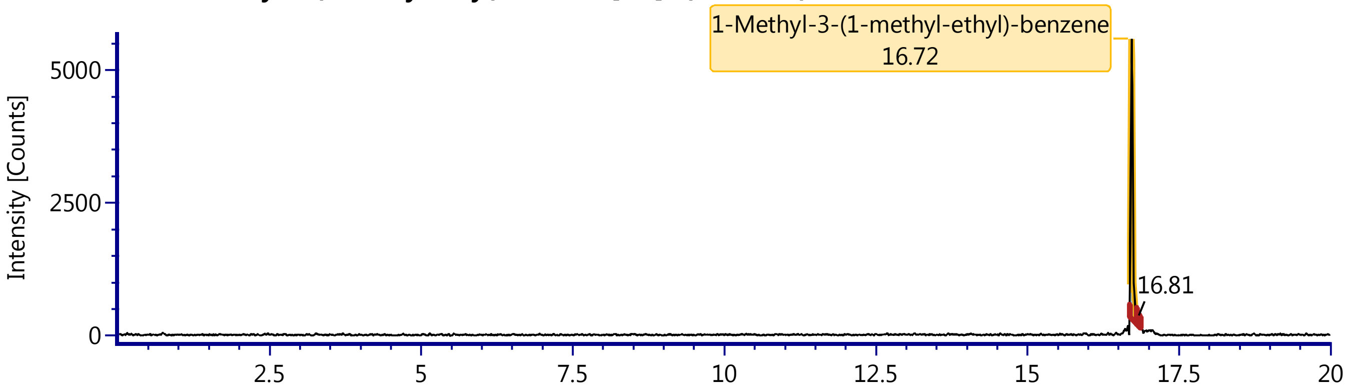

Item name: 230526_305 R 93_1_Pos
Channel name: Bakuchiol [+H] : (33.3 PPM) 257.1886

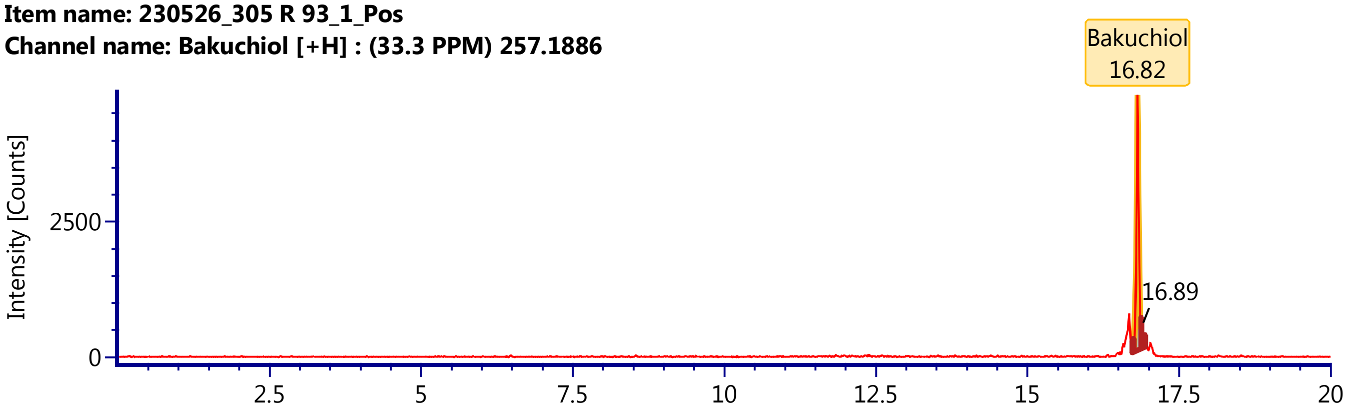

Item name: 230526_305 R 93_1_Pos
Channel name: Curculigo saponin A [+H] : (33.3 PPM) 637.4285

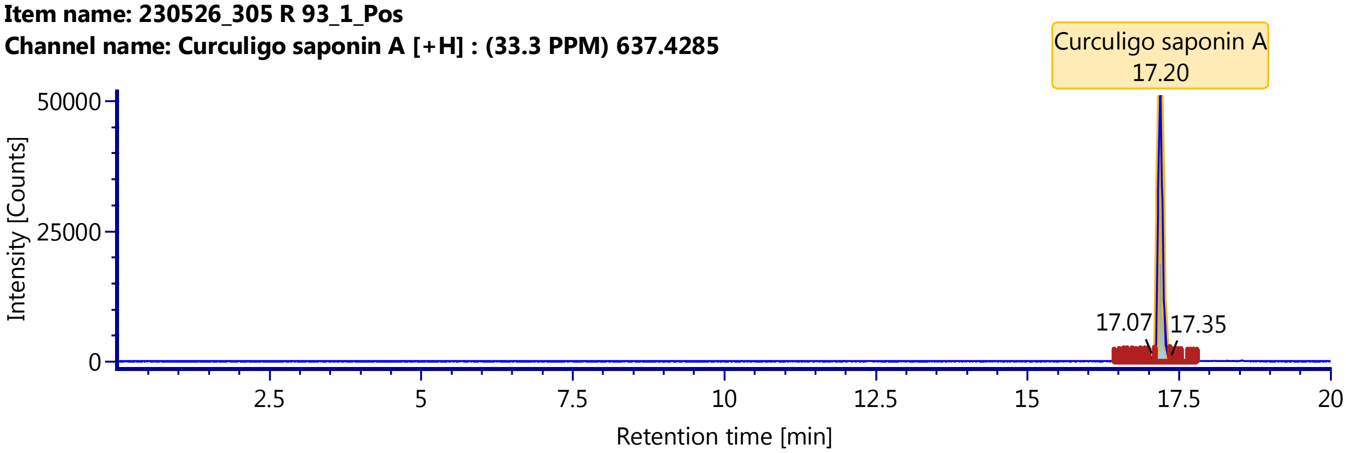

Item name: 230526_305 R 93_1_Pos
Channel name: Dehydrolindestrenolide [+H] : (33.3 PPM) 229.1217

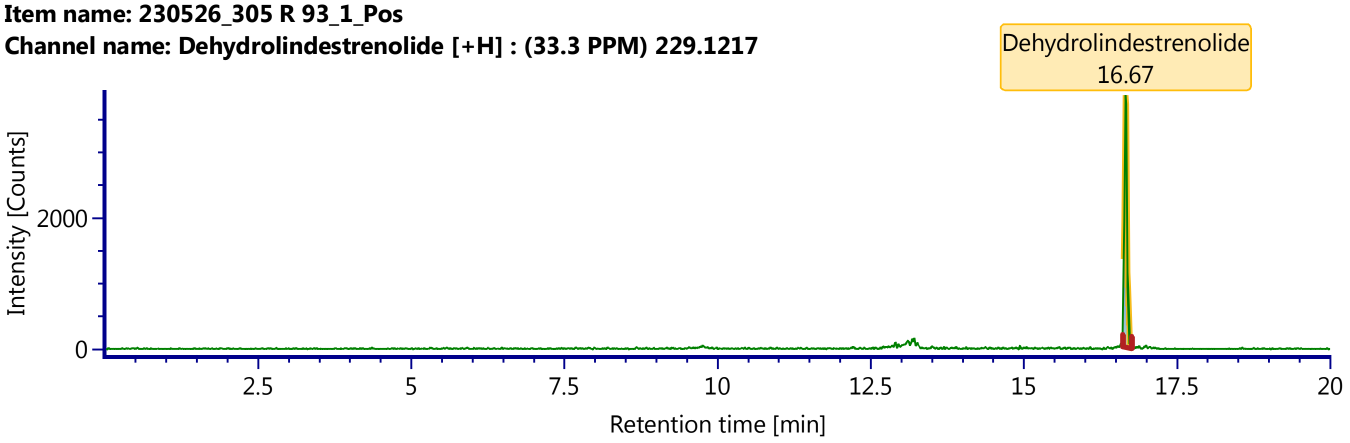

Item name: 230526_305 R 93_1_Pos **Component name: 1-Methyl-3-(1-methyl-ethyl)-benzene**
Item description:

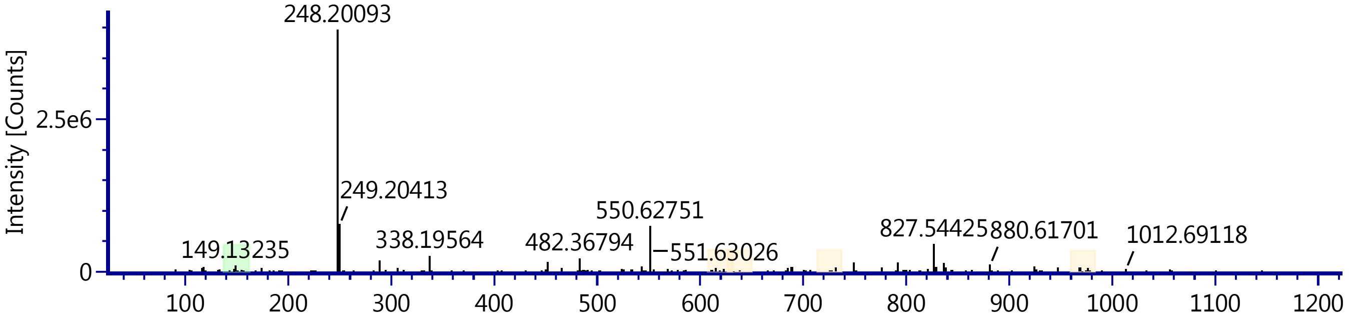

Item name: 230526_305 R 93_1_Pos **Component name: 1-Methyl-3-(1-methyl-ethyl)-benzene**
Item description:

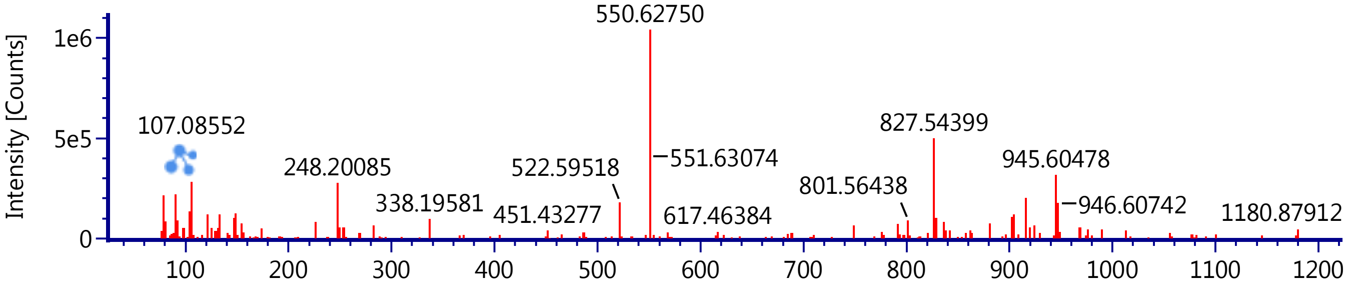

Item name: 230526_305 R 93_1_Pos **Component name: Bakuchiol**
Item description:

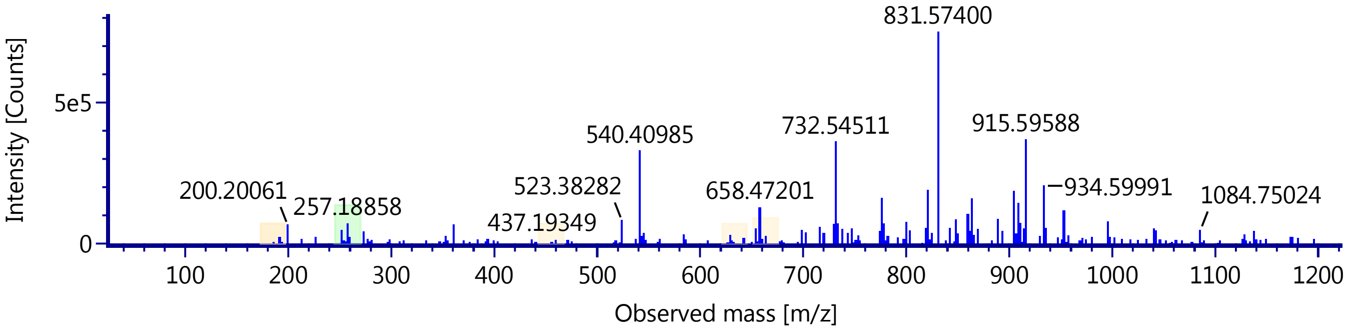

Item name: 230526_305 R 93_1_Pos **Component name: Bakuchiol**
Item description:

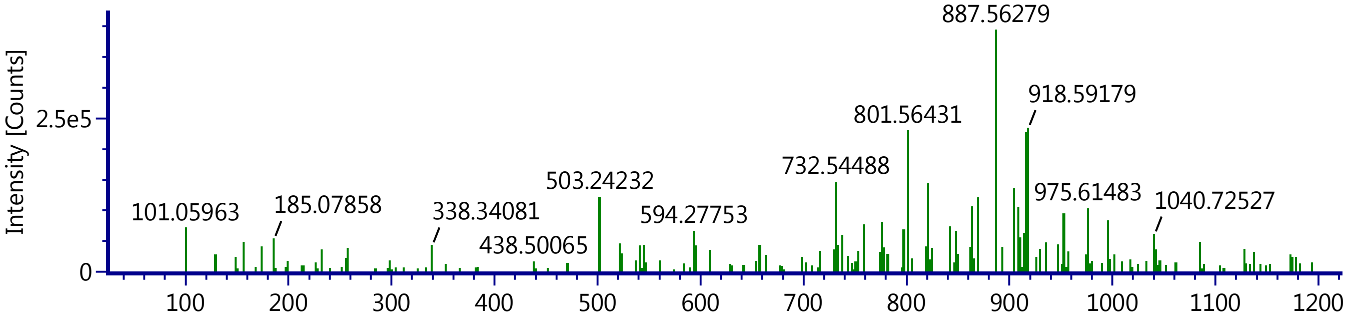

Item name: 230526_305 R 93_1_Pos **Component name: Curculigo saponin A**
Item description:

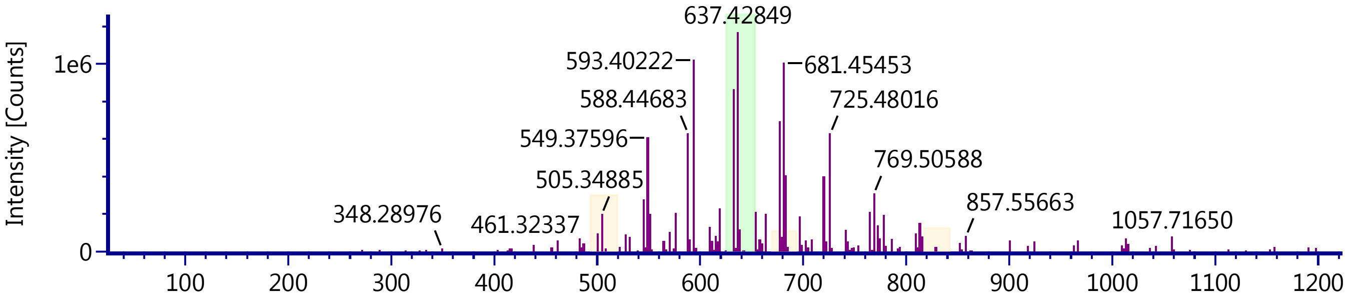

Item name: 230526_305 R 93_1_Pos **Component name: Curculigo saponin A**
Item description:

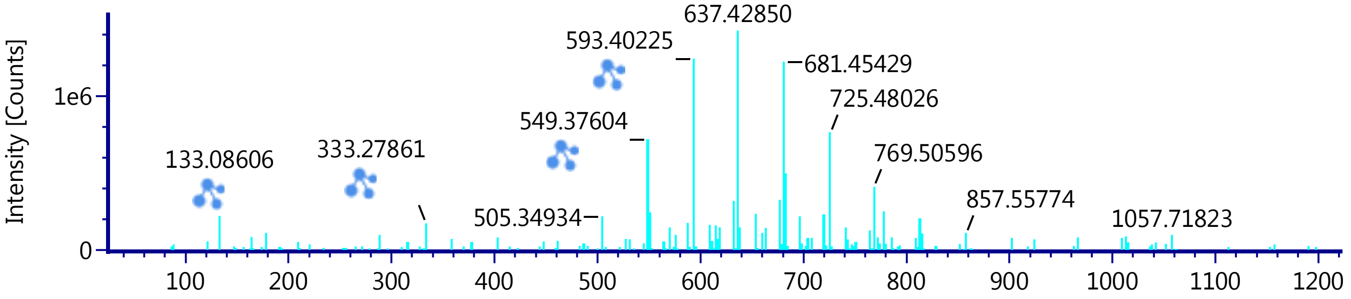

Item name: 230526_305 R 93_1_Pos **Component name: Dehydrolindestrenolide**
Item description:

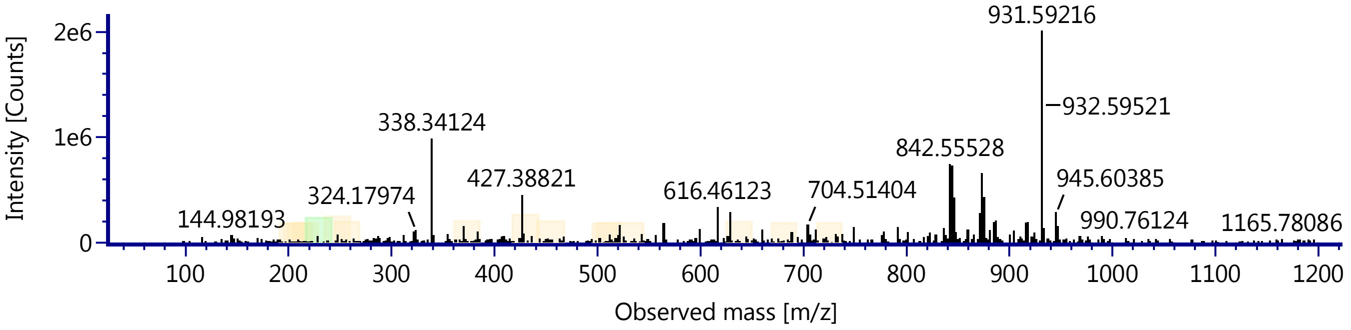

Item name: 230526_305 R 93_1_Pos
Item description:

Component name: Dehydrolindestrenolide

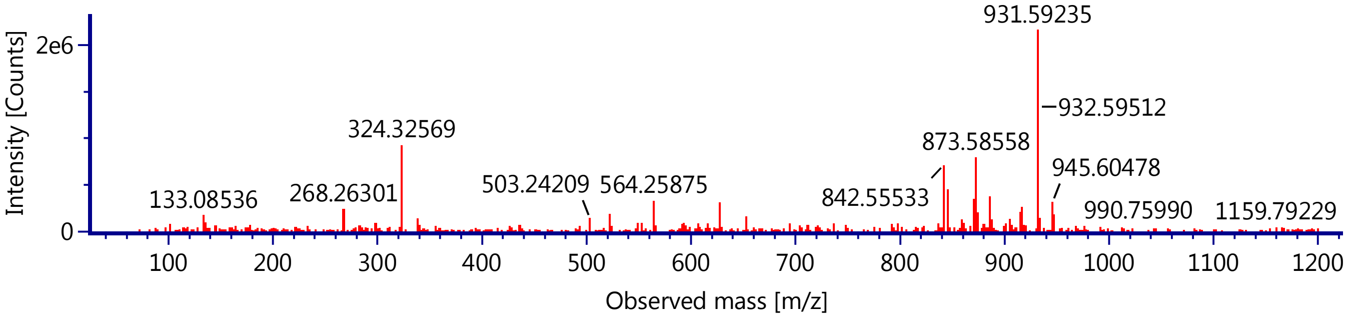

The BPI Plot of Sampel

Item name: 230526_305 R 93_2_Pos
Channel name: 1: TOF MS^E BPI (50-1200) 6eV ESI+ : Integrated : Smoothed

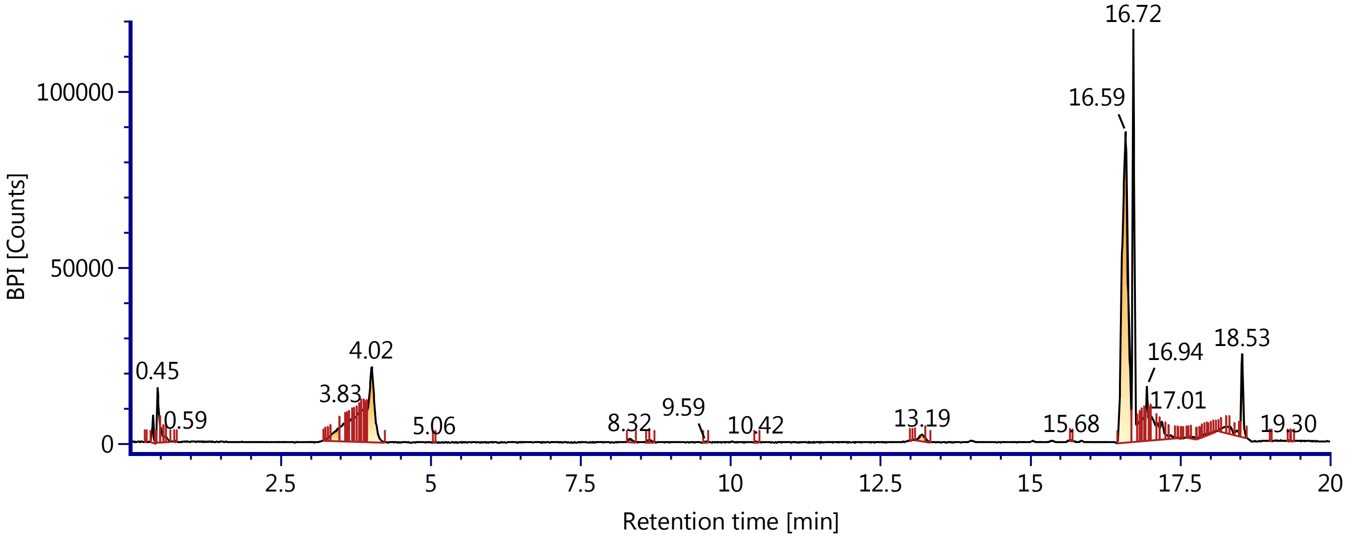

Component Plot

Item name: 230526_305 R 93_2_Pos

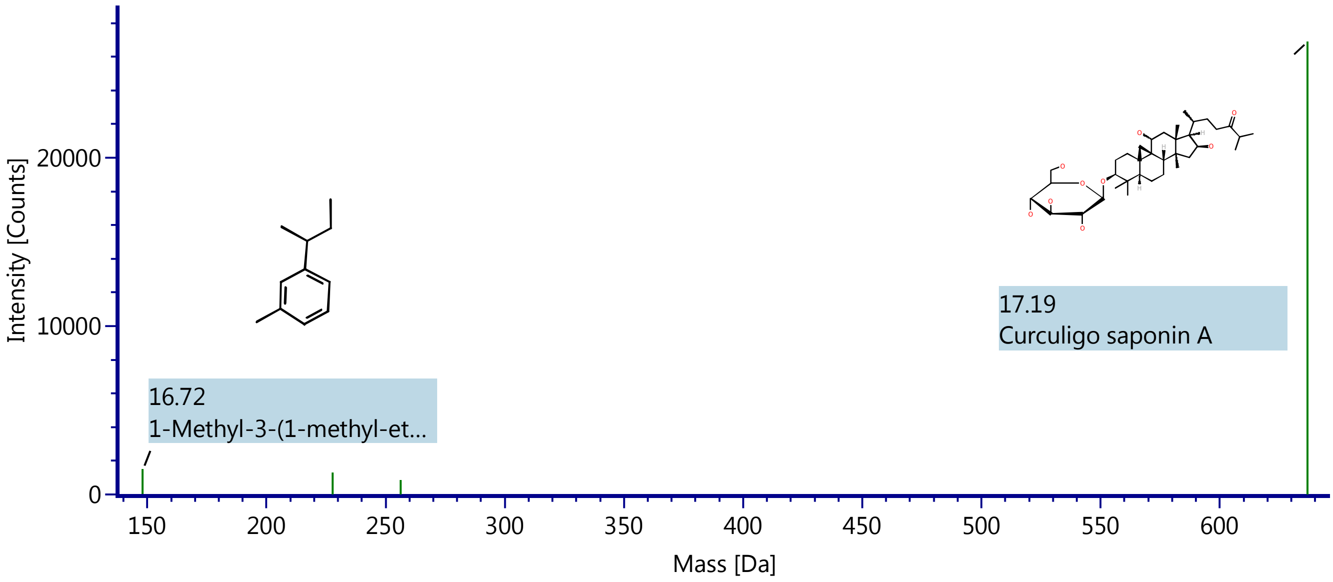

Component Summary (Confirmed)

Confirmed Component

Component name	Formula	Identification status	Observed RT (min)	Mass error (ppm)	Total Fragments Found	Isotope Match Mz RMS PPM	Isotope Match Intensity RMS Percent	Response	Adducts
1-Methyl-3-(1-methyl-ethyl)-benzene	C11H16	Identified	16.72	-1.4	12	1.58	2.41	1493	+H
Bakuchiol	C18H24O	Identified	16.82	0.7	2	0.90	2.93	817	+H
Curculigo saponin A	C36H60O9	Identified	17.19	-3.9	19	4.47	2.13	26866	+H
Dehydrolindestrenolide	C15H16O2	Identified	16.67	-1.4	17	3.32	3.19	1272	+H

Item name: 230526_305 R 93_2_Pos
Channel name: 1-Methyl-3-(1-methyl-ethyl)-benzene [+H] : (33.3 PPM) 149.1323

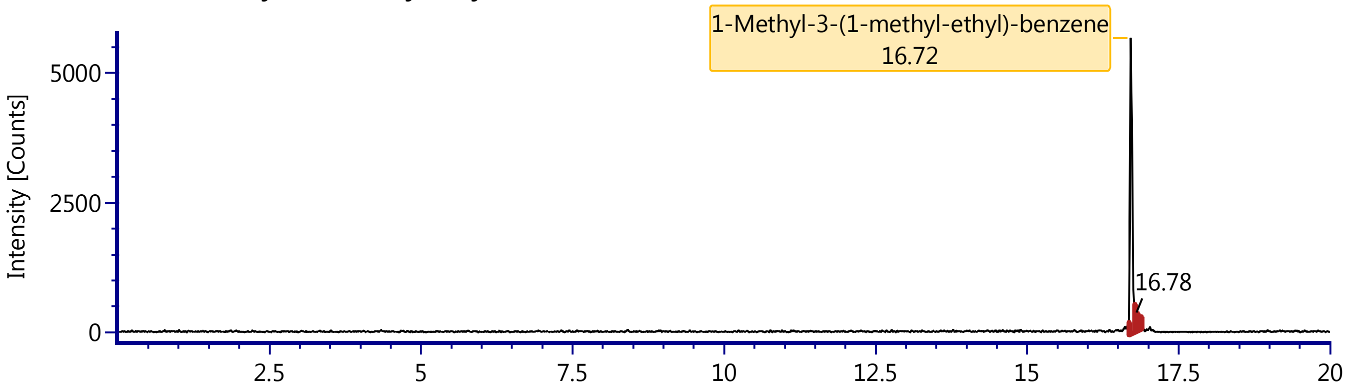

Item name: 230526_305 R 93_2_Pos
Channel name: Bakuchiol [+H] : (33.3 PPM) 257.1902

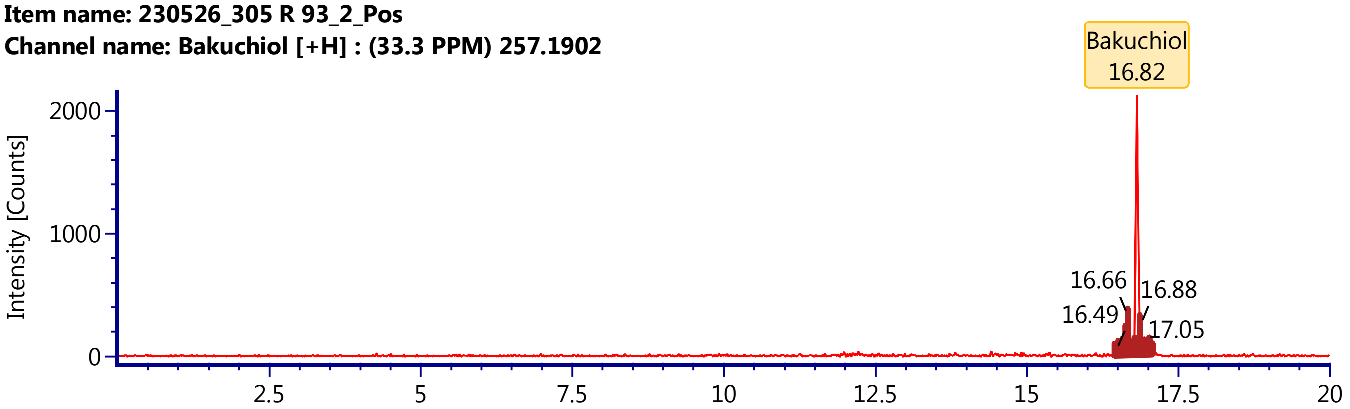

Item name: 230526_305 R 93_2_Pos
Channel name: Curculigo saponin A [+H] : (33.3 PPM) 637.4285

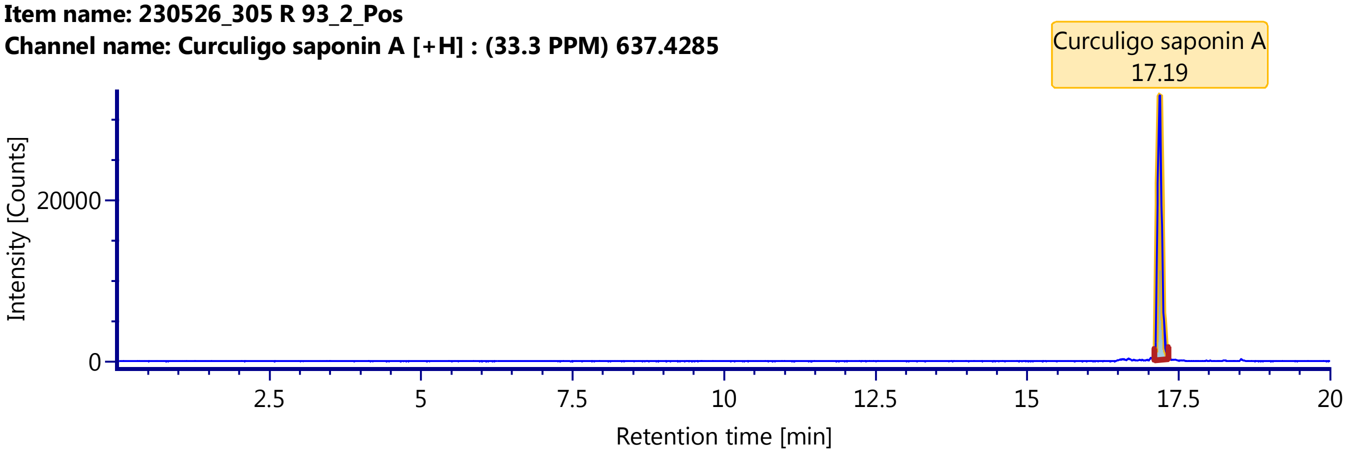

Item name: 230526_305 R 93_2_Pos
Channel name: Dehydrolindestrenolide [+H] : (33.3 PPM) 229.1220

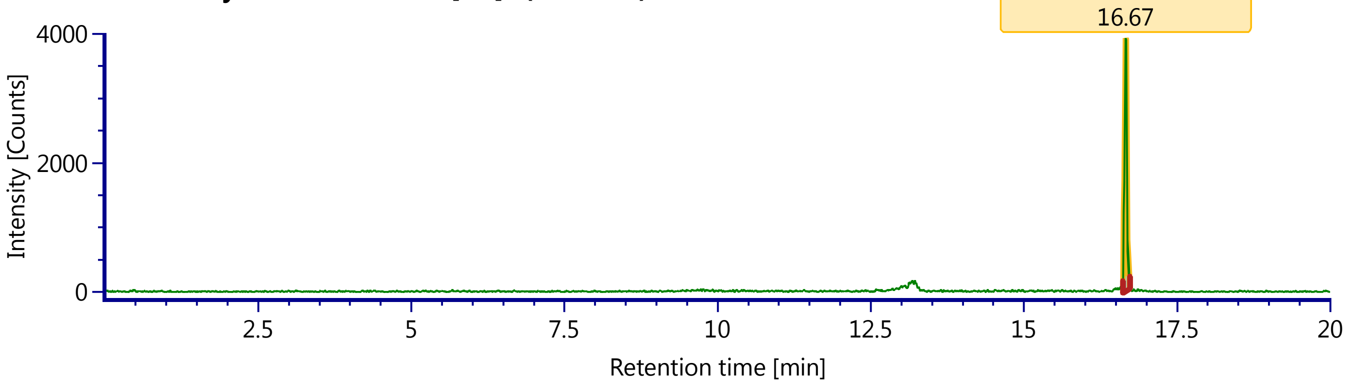

Item name: 230526_305 R 93_2_Pos **Component name: 1-Methyl-3-(1-methyl-ethyl)-benzene**
Item description:

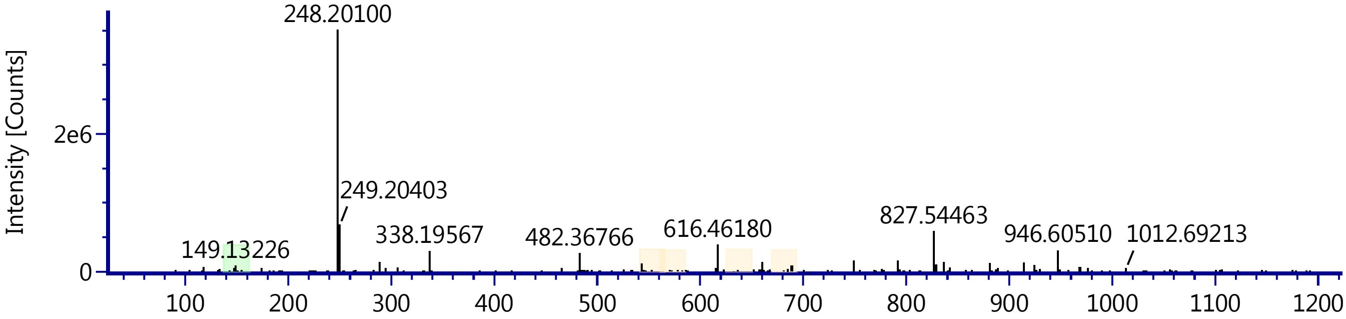

Item name: 230526_305 R 93_2_Pos **Component name: 1-Methyl-3-(1-methyl-ethyl)-benzene**
Item description:

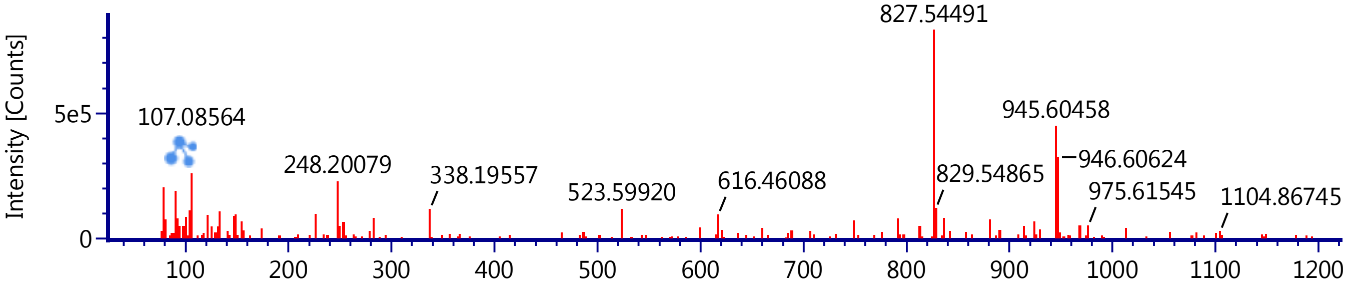

Item name: 230526_305 R 93_2_Pos **Component name: Bakuchiol**
Item description:

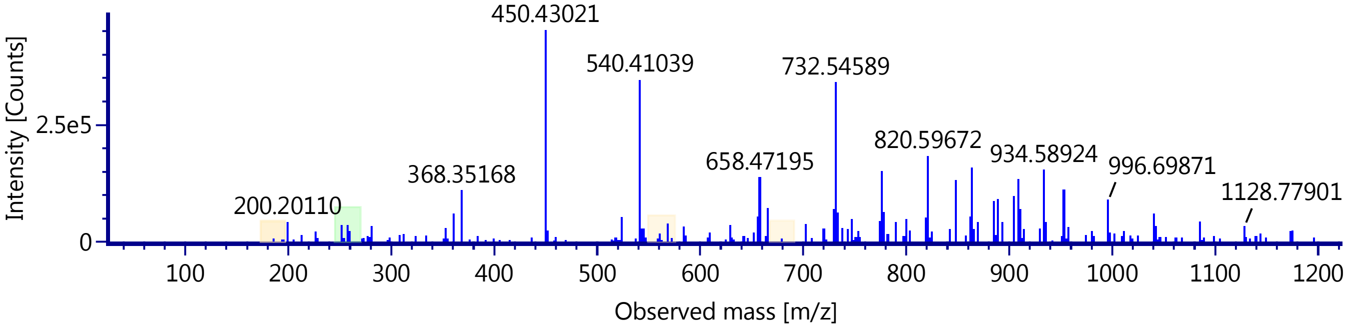

Item name: 230526_305 R 93_2_Pos **Component name: Bakuchiol**
Item description:

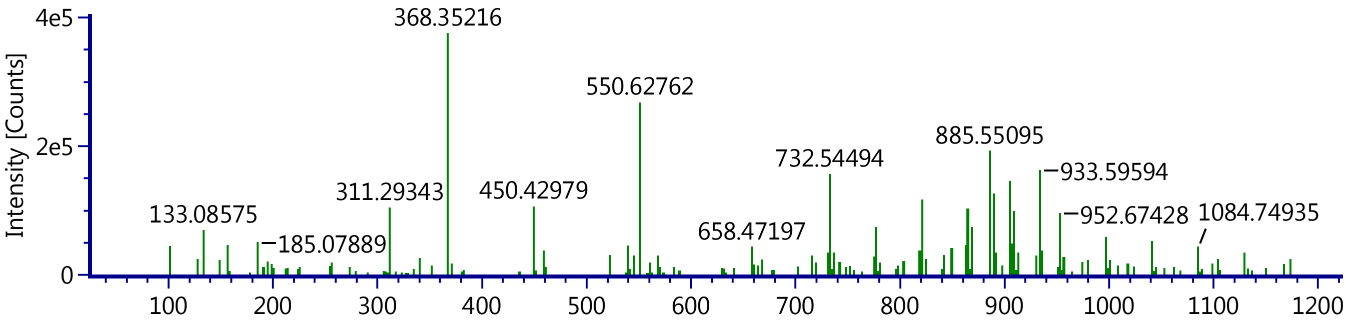

Item name: 230526_305 R 93_2_Pos **Component name: Curculigo saponin A**
Item description:

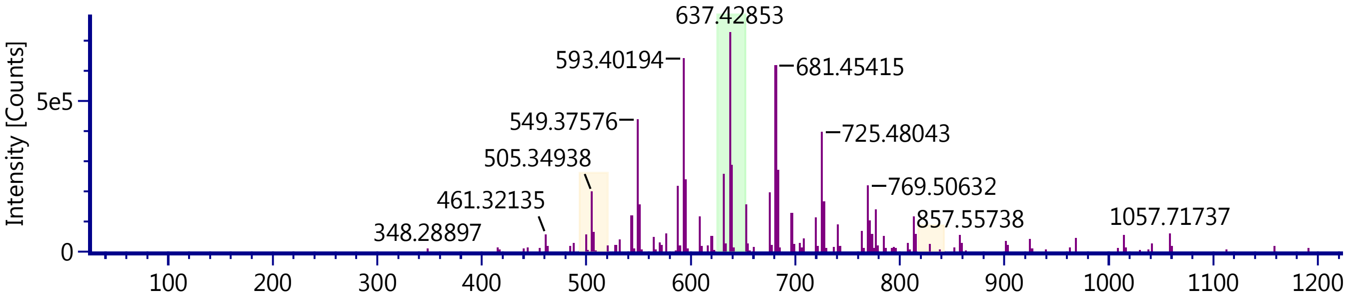

Item name: 230526_305 R 93_2_Pos **Component name: Curculigo saponin A**
Item description:

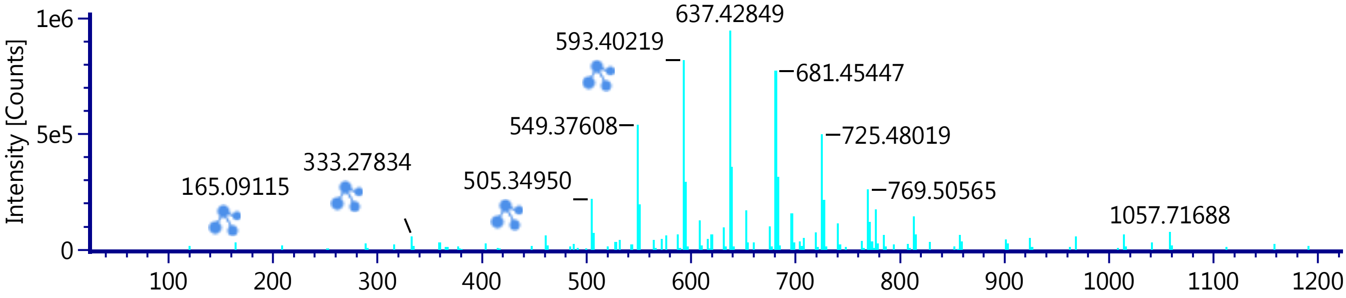

Item name: 230526_305 R 93_2_Pos **Component name: Dehydrolindestrenolide**
Item description:

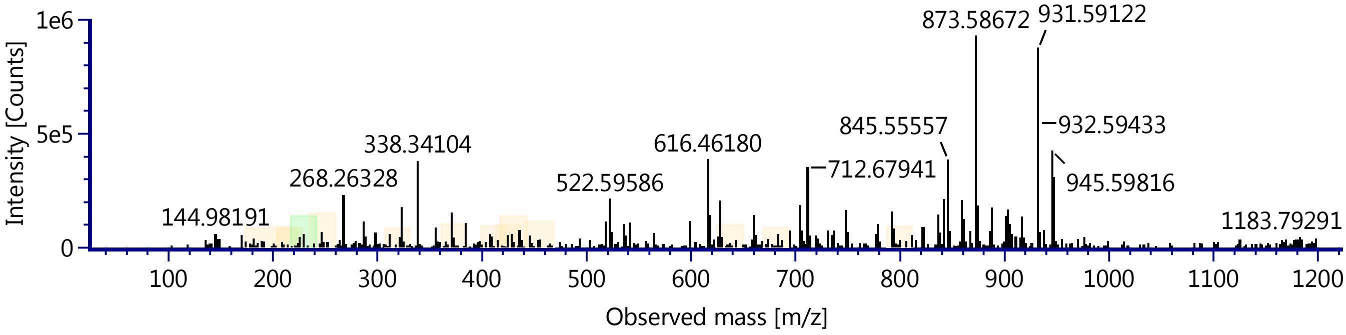

Item name: 230526_305 R 93_2_Pos **Component name: Dehydrolindestrenolide**
Item description:

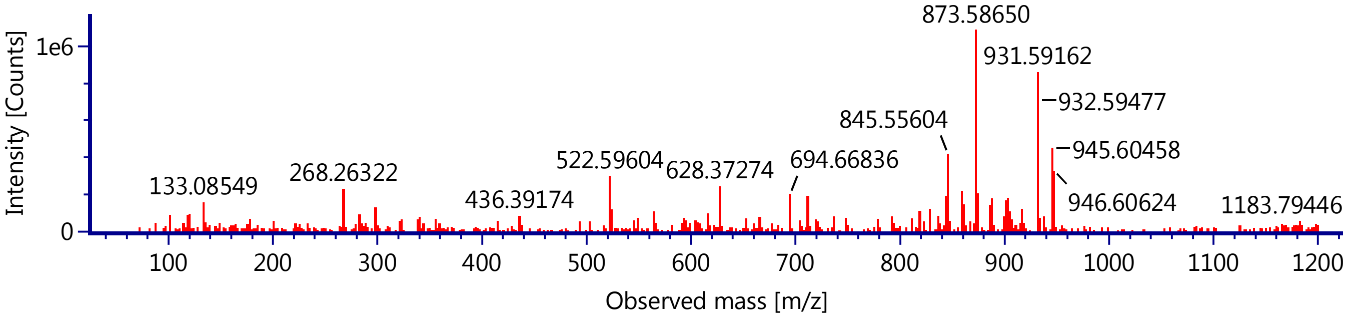

Supplement: Supplementary file 1 [file nutrients-15-03886-s001.zip › Supplementary S1 EBN - 230605 NP (305.R.93) Kromatogram ESI +.pdf]
