# Supplementary material for: Revealing Edible Bird Nest as Novel Functional Foods in Combating Metabolic Syndrome: Comprehensive In Silico, In Vitro, and In Vivo Studies
Source: Nutrients. 2023 Sep 6;15(18):3886. doi: 10.3390/nu15183886 (PMC10535673; doi:10.3390/nu15183886)
Supplement: Supplementary file 1 [file nutrients-15-03886-s001.zip › Supplementary S2. Visualisasi Docking EBN.pdf]

iNOS 3E7G

No.	Substance	Ligand Interaction
Native Ligand		
1	3E7G	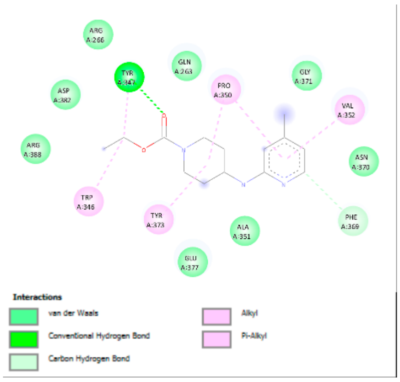
Control		
1	S-ibuprofen	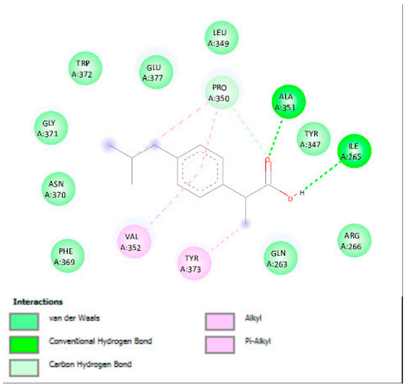
Ligan Uji		
1	Bakuchiol	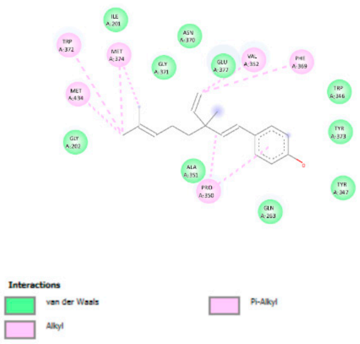

2	Curculigosaponin A	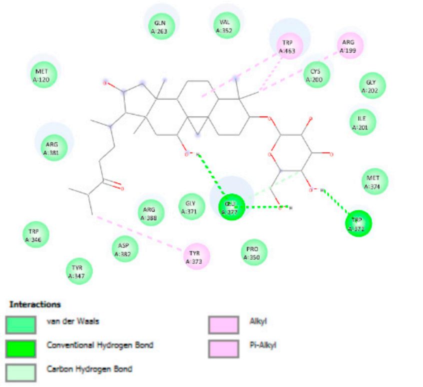
3	Dehydrolindestrenolide	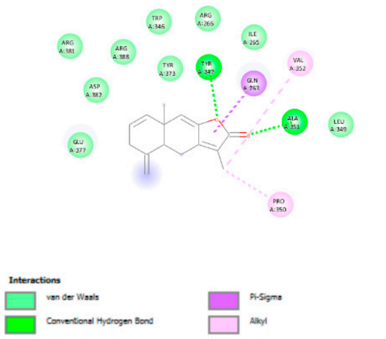
4	1-Methyl-3-(1-methyl-ethyl)-benzene	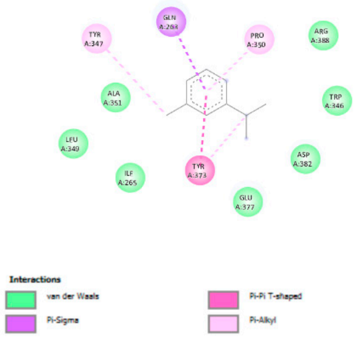

ROS1 Kinase 3ZBF

No.	Substance	Ligand Interaction
Native Ligand		

<table>
<tr><td>1</td><td>3ZBF</td><td>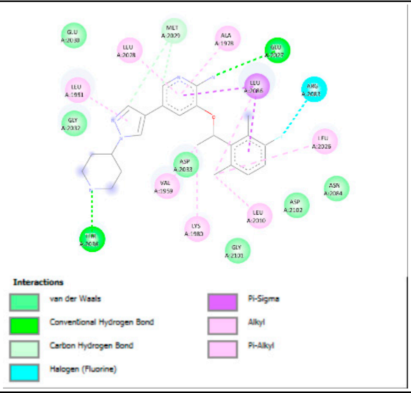
</td></tr>
<tr><td colspan="2">Control</td><td></td></tr>
<tr><td>1</td><td>Trolox</td><td>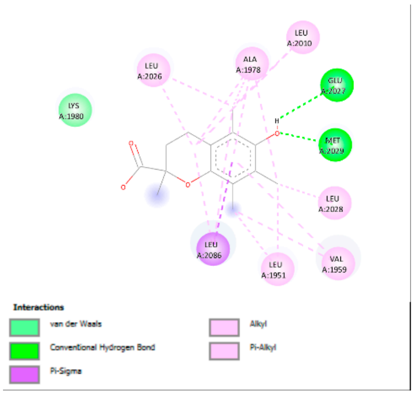
</td></tr>
<tr><td colspan="2">Ligan Uji</td><td></td></tr>
<tr><td>1</td><td>Bakuchiol</td><td>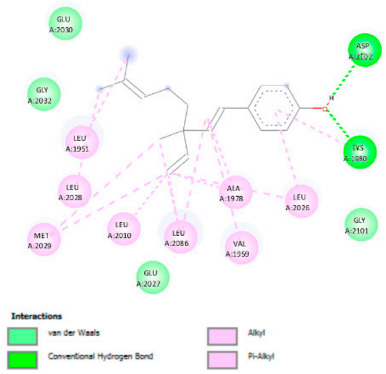
</td></tr>
<tr><td>2</td><td>Curculigosaponin A</td><td>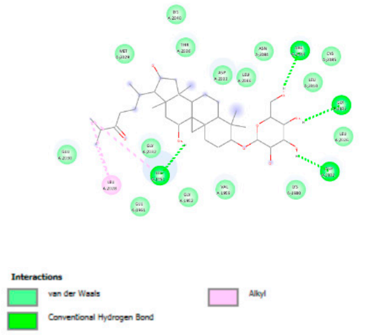
</td></tr>
</table>

3	Dehydrolindestrenolide	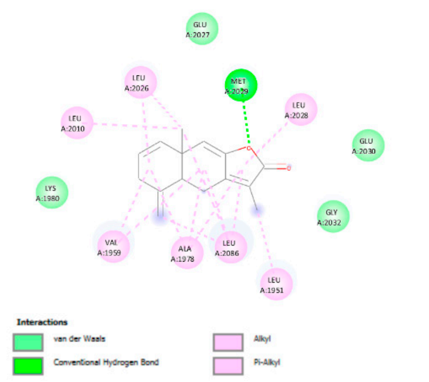
4	1-Methyl-3-(1-methyl-ethyl)-benzene	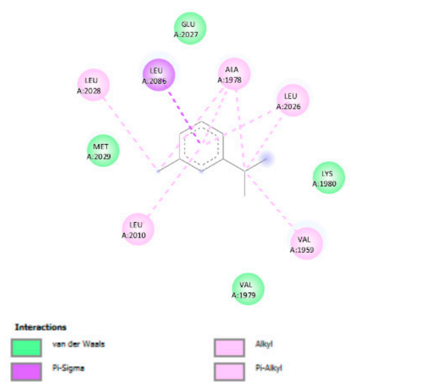

human pancreatic lipase 1LPB

No.	Substance	Ligand Interaction
Native Ligand		
1	1LPB	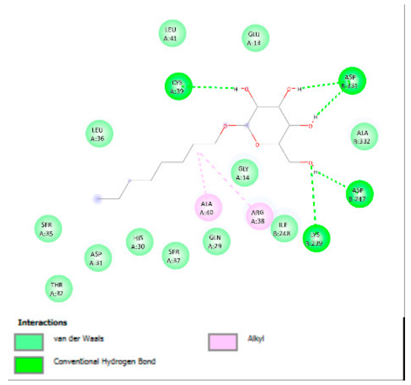
Control		

1	Orlistat	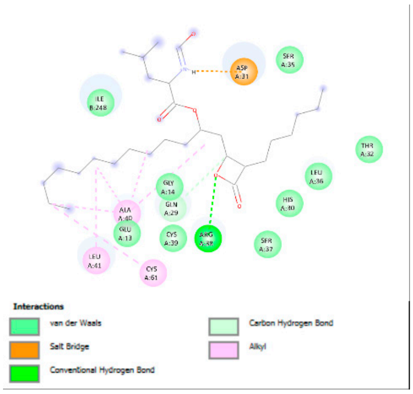
Ligan Uji		
1	Bakuchiol	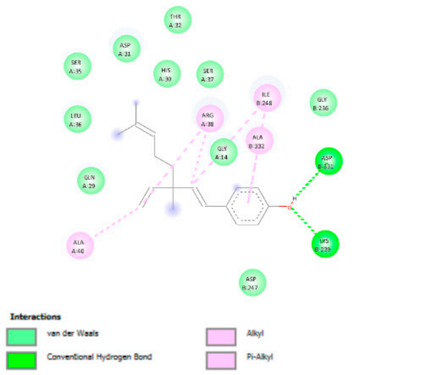
2	Curculigosaponin A	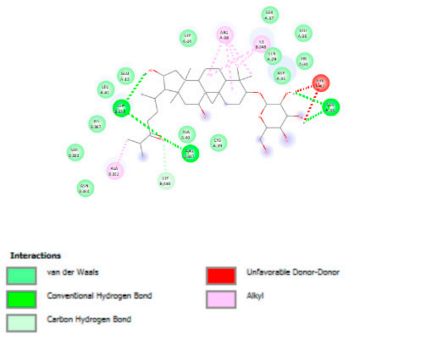
3	Dehydrolindestrenolide	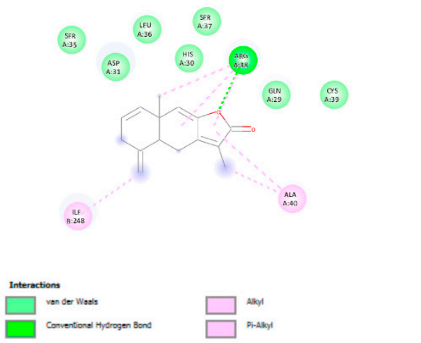

4	1-Methyl-3-(1-methyl-ethyl)-benzene	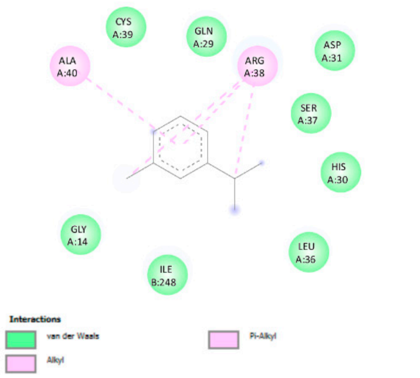

Fat mass and obesity-associated (FTO) protein 3LFM

No.	Substance	Ligand Interaction
Native Ligand		
1	3LFM	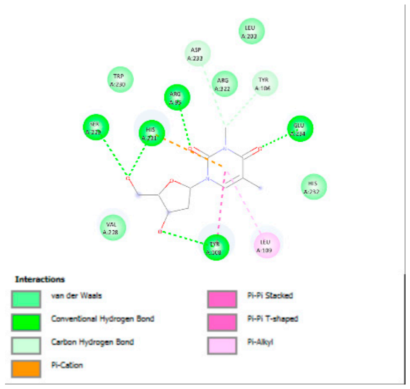
Control		
1	Orlistat	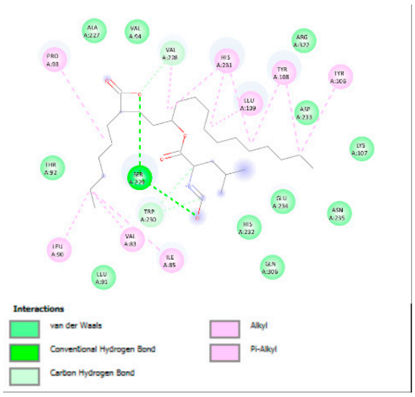
Ligan Uji		

<table>
<tr><td>1</td><td>Bakuchiol</td><td>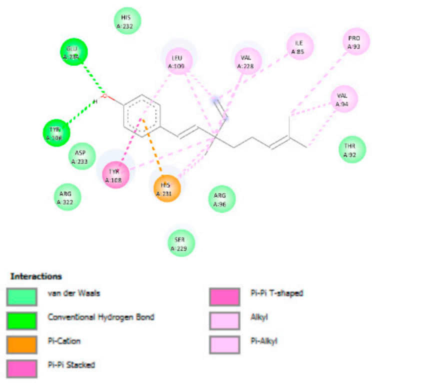
</td></tr>
<tr><td>2</td><td>Curculigosaponin A</td><td>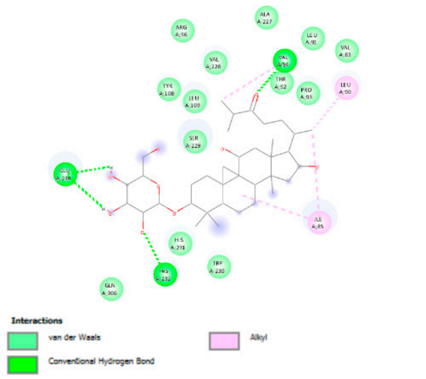
</td></tr>
<tr><td>3</td><td>Dehydrolindestrenolide</td><td>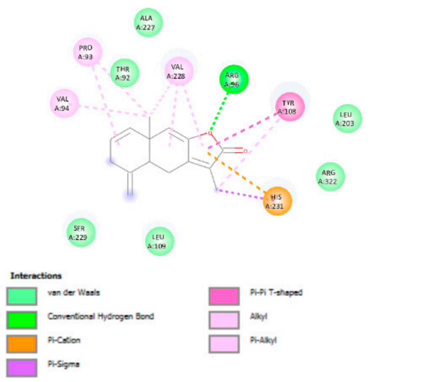
</td></tr>
<tr><td>4</td><td>1-Methyl-3-(1-methyl-ethyl)-benzene</td><td>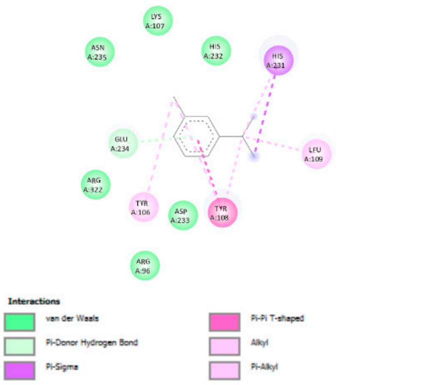
</td></tr>
</table>
